# Phylum-Spanning Neuropeptide GPCR Identification and Prioritization: Shaping Drug Target Discovery Pipelines for Nematode Parasite Control

**DOI:** 10.3389/fendo.2021.718363

**Published:** 2021-09-30

**Authors:** Louise E. Atkinson, Ciaran J. McCoy, Bethany A. Crooks, Fiona M. McKay, Paul McVeigh, Darrin McKenzie, Allister Irvine, John Harrington, Bruce A. Rosa, Makedonka Mitreva, Nikki J. Marks, Aaron G. Maule, Angela Mousley

**Affiliations:** ^1^ Microbes and Pathogen Biology, The Institute for Global Food Security, School of Biological Sciences, Queen’s University Belfast, Belfast, United Kingdom; ^2^ Boehringer Ingelheim Animal Health, Athens, GA, United States; ^3^ Division of Infectious Diseases, Department of Medicine, Washington University School of Medicine, St. Louis, MO, United States; ^4^ McDonnell Genome Institute, Washington University School of Medicine, St. Louis, MO, United States

**Keywords:** neuropeptide, G-protein coupled receptor, FMRF-amide like peptide, drug target, nematode parasite

## Abstract

Nematode parasites undermine human health and global food security. The frontline anthelmintic portfolio used to treat parasitic nematodes is threatened by the escalation of anthelmintic resistance, resulting in a demand for new drug targets for parasite control. Nematode neuropeptide signalling pathways represent an attractive source of novel drug targets which currently remain unexploited. The complexity of the nematode neuropeptidergic system challenges the discovery of new targets for parasite control, however recent advances in parasite ‘omics’ offers an opportunity for the *in silico* identification and prioritization of targets to seed anthelmintic discovery pipelines. In this study we employed Hidden Markov Model-based searches to identify ~1059 *Caenorhabditis elegans* neuropeptide G-protein coupled receptor (*Ce-NP-GPCR*) encoding gene homologs in the predicted protein datasets of 10 key parasitic nematodes that span several phylogenetic clades and lifestyles. We show that, whilst parasitic nematodes possess a reduced complement of *Ce-NP-GPCRs*, several receptors are broadly conserved across nematode species. To prioritize the most appealing parasitic nematode NP-GPCR anthelmintic targets, we developed a novel *in silico* nematode parasite drug target prioritization pipeline that incorporates pan-phylum *NP-GPCR* conservation, *C. elegans-*derived reverse genetics phenotype, and parasite life-stage specific expression datasets. Several NP-GPCRs emerge as the most attractive anthelmintic targets for broad spectrum nematode parasite control. Our analyses have also identified the most appropriate targets for species- and life stage- directed chemotherapies; in this context we have identified several *NP-GPCRs* with macrofilaricidal potential. These data focus functional validation efforts towards the most appealing NP-GPCR targets and, in addition, the prioritization strategy employed here provides a blueprint for parasitic nematode target selection beyond NP-GPCRs.

## Introduction

Nematode parasites continue to have a global impact on human health and agricultural productivity such that novel mode-of-action anthelmintics are critical for sustained parasite control, especially in light of the escalation in anthelmintic resistance (1–3). Whilst the nematode neuromuscular system is a proven drug target, it remains underexploited (4, 5). Indeed, the majority of frontline anthelmintics only target aspects of neuromuscular signaling controlled by ion channels, however the neuropeptide signaling system is also critical to normal nematode neuromuscular function (6, 7).

Within the neuromuscular signalling system, neuropeptide GPCRs (NP-GPCRs) have been identified as highly ‘druggable’ targets (8). Indeed, an estimated 34% of human drugs act on GPCRs (9, 10). Despite this, NP-GPCRs have yet to be exploited for chemotherapeutic control of nematode parasites. In part, this is due to limited knowledge of NP-GPCR profiles in key parasitic nematode species which would enable NP-GPCR target prioritization.

Recent advances in ‘omics’ technologies have driven a significant expansion of *in silico* data for parasitic nematodes (11), providing an opportunity for the identification of novel putative anthelmintic targets. However, the volume and complexity of the available datasets presents a significant challenge to target prioritization. *In silico* prioritization approaches are essential given the lack of tractable and scalable reverse genetics tools for parasitic nematode systems (12, 13).

Analysis of the *Caenorhabditis elegans* genome suggests the presence of 152 putative *NP-GPCRs* (14), several of which are likely to represent attractive and exploitable anthelmintic targets. Indeed, functional studies indicate that some *Ce*-*NP-GPCR* knockdown/knockout worms display aberrant phenotypes that include paralysis and death [see WormBase; Harris et al. (15)]. Despite this, we have limited knowledge of NP-GPCR encoding gene conservation and life-stage expression in therapeutically relevant parasitic nematodes. These data are essential to drive the prioritization of parasite NP-GPCR drug targets for functional validation and chemotherapeutic exploitation.

In this study we employed *in silico* approaches to: (i) characterise the *NP-GPCR* complements of 10 key parasitic nematode species; (ii) develop a novel nematode drug target prioritization pipeline that incorporates *NP-GPCR* conservation, expression and functional data, and (iii) identify NP-GPCRs that represent putative, novel, broad spectrum parasitic nematode control targets. Integration of these multi-omics-derived datasets provides a springboard for functional biology that will improve our understanding of fundamental nematode neurosignalling and support future anthelmintic discovery efforts.

## Materials And Methods

### Putative NP-GPCR Identification

**Figure 1:**
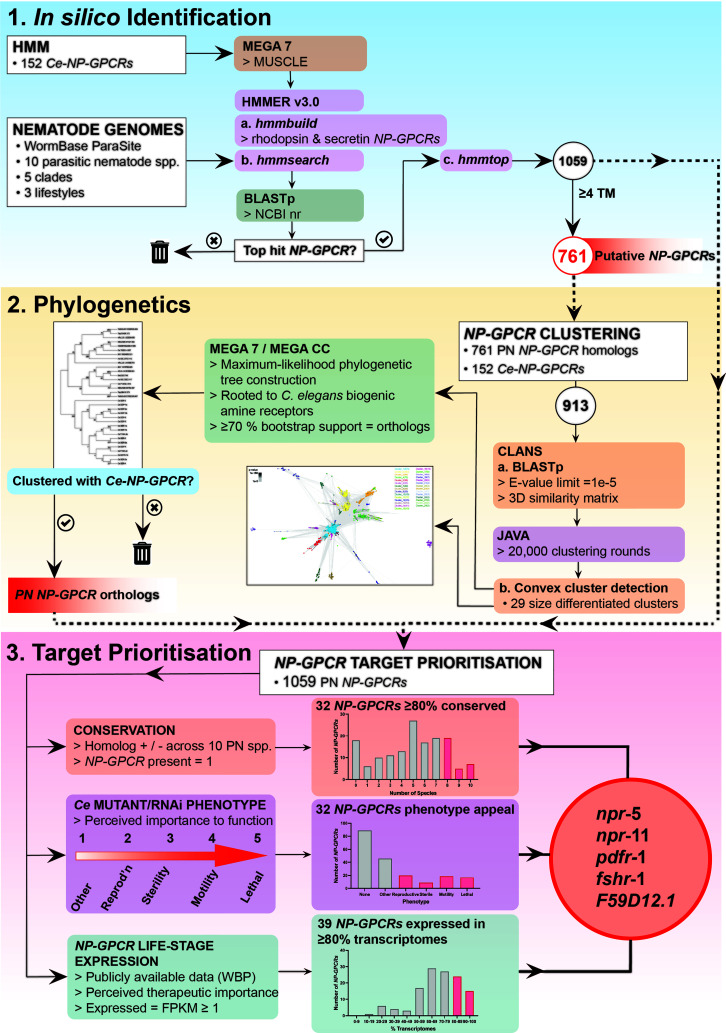
NP-GPCR drug target prioritization pipeline demonstrating *NP-GPCR* identification, phylogenetics and target prioritization workflow and a summary of the data generated at key stages in the pipeline. Five parasitic nematode *NP-GPCRs* are prioritised for validation in parasites: *npr*-5, -11, *pdfr*-1, *fshr*-1 and *F59D12.1*. HMM, Hidden Markov Models; TM, transmembrane domains; PN, parasitic nematode; CLANS, Cluster Analysis of Sequences; Ce, *Caenorhabditis elegans*; Reprod’n, reproduction; EXP, expression; WBP, WormBase Parasite; KO, knockout; RNAi, RNA interference.

Putative nematode *NP-GPCRs* were identified *via* multiple sequence alignment derived Hidden Markov Models (HMMs), using methods based on those previously described (16). Briefly, HMMs were constructed using predicted protein alignments of all putative *C. elegans* neuropeptide receptors (14). Alignments were generated using MEGA 7 with default MUSCLE settings (17). Distinct models were constructed with rhodopsin and secretin NP-GPCR family members (14) using default *hmmbuild* parameters [HMMER v3; Mistry et al. (18)]. *hmmsearch* (HMMER v3) was employed to identify potential *NP-GPCRs* within the predicted protein datasets of 10 phylogenetically dispersed nematode parasites (*Trichuris muris, Trichinella spiralis, Romanomermis culicivorax, Ascaris suum, Brugia malayi, Dirofilaria immitis, Necator americanus, Haemonchus contortus, Bursaphelenchus xylophilus, Globodera pallida;* see (**Supplementary Table 1**), using default settings. The putative *NP-GPCR* sequences identified *via hmmsearch* were then used as queries in BLASTp searches in the NCBI non-redundant database (https://blast.ncbi.nlm.nih.gov; default settings) to identify the most similar sequences in *C. elegans*. Queries that failed to return a putative *NP-GPCR* as the highest scoring pair/top hit were excluded from downstream analyses. Putative *NP-GPCR* sequences were then filtered based on the number of transmembrane (TM) domains, as predicted by *hmmtop* (19). Returns containing 4 or more TM regions were excluded from downstream phylogenetic analyses (see **Supplementary Figure 1** for a species-specific summary of the TM domain composition of all returns present in the putative *NP-GPCR* datasets), but still included in the drug target prioritization pipeline (see **Figure 1**).

### *NP-GPCR* Clustering and Phylogenetic Analyses

The CLANS algorithm (https://toolkit.tuebingen.mpg.de/#/tools/clans) was used to identify convex clusters within the *NP-GPCR* datasets (20). Parasite *NP-GPCR* hits (761 putative parasite *NP-GPCR* sequences with ≥4 TM domains) were analysed alongside all putative *NP-GPCRs* from *C. elegans* (14). *NP-GPCR* sequences were uploaded to the CLANS website; BLAST high scoring pairs were extracted up to an E-value limit of 1e-5, all other parameters remained at default. CLANS performed a series of all-against-all BLASTp comparisons between every sequence submitted, generating a 3D similarity matrix constructed from the e-values of each individual search. The CLANS file output was visualized and coloured after 20,000 clustering rounds using the Java-based desktop software. CLANS convex cluster detection algorithm was used to delineate clusters of sequences under default settings. Clusters were numbered according to size (Cluster 1 being the largest). Individual clusters were used in Maximum-likelihood phylogenetic tree construction using MEGA 7 or MEGA CC (17), depending on the computing requirements of individual trees. Note that, where CLANS delineated clusters within the previously defined NP-GPCR families (14), these clusters were amalgamated prior to further phylogenetic analyses. Similarly, satellite singleton (non-clustered) sequences and small clusters that lacked any putative *C. elegans* homolog were grouped with their nearest-neighbour cluster prior to tree construction. Sequences extracted from each cluster were aligned *via* default MUSCLE settings in MEGA 7. Alignments were analyzed using the ‘find best DNA/Protein Models (ML)’ option to determine the most appropriate model of evolution for tree construction. All trees were constructed using: the bootstrap method (500 replicates); the LG model of evolution (G+I) with 5 discrete Gamma categories; a partial deletion of gaps (80% site coverage cut-off); and the nearest-neighbour interchange algorithm with no branch swap filter. Trees were rooted using a selection of *C. elegans* biogenic amine receptor sequences (see **Supplementary Figures 2–12**). Returns that clustered with a specific *Ce-NP-GPCR* with ≥70% bootstrap support were considered orthologs. Where returns failed to cluster with a specific *Ce-NP-GPCR*, but clustered with multiple *C. elegans* paralogs within the same NP-GPCR family, they were assigned based on top BLAST hit.

### Drug Target Prioritization

A drug target prioritization pipeline based on: (i) NP-GPCR-encoding gene conservation (generated in this study); (ii) *C. elegans* derived functional data (15), and (iii) publicly available RNASeq data (see **Supplementary Table 1**), were collated and curated as outlined in **Figure 1**. Briefly, *NP-GPCR* conservation profiles across the nine key parasite species in this study were analysed using the phylogenetics approach described above. To enable the inclusion of all putative *NP-GPCR* hits in the prioritization pipeline, and to circumvent prioritization bias by losing those that possess partial sequence availability (<4 TM domains; not suitable for phylogenetic analyses), predicted proteins with <4 TM regions were included as homologs of the highest scoring *C. elegans* BLAST hit.

Phenotype data associated with *C. elegans* mutant/RNAi experiments for the 152 known *NP-GPCRs* were collated from observed phenotypes reported on WormBase [version WS280; Harris et al. (15)]. Each *Ce*-NP-GPCR encoding gene was scored based on phenotype significance (with relevance to anthelmintic target discovery), where no recorded phenotype scored 0, reproductive scored 2, sterility scored 3, motility scored 4, and lethality scored 5. Any other recorded phenotype scored 1. Many *Ce*-NP-GPCR encoding genes had multiple phenotypes recorded; in this scenario, phenotype scores were combined to provide an overall total phenotype score for each *NP-GPCR*. Where multiple phenotypes within the same category were recorded, the category was only scored once.

RNASeq data were accessed from published life-stage specific transcriptome datasets [untreated/wildtype: *T. muris* (21); *A. suum* (22, 23); *B. malayi* (24, 25); *D. immitis* (26); *H. contortus* (27), and *G. pallida* (28)]. An FPKM value of 1 was used as the threshold for transcript expression (where FPKM ≥1 was deemed to be expressed). RNASeq data for *H. contortus* and *T. muris* (raw counts and FPKM) were generated following an established RNASeq pipeline. Raw sequences reads [PRJEB1360 (27); PRJEB1054 (21)] were downloaded and split into forward and reverse fastq files using NCBI SRA Toolkit (29). Reads were trimmed using Trimmomatic [v0.36; parameter: LEADING:5 TRAILING:5 SLIDINGWINDOW:3:15 MINLEN:34 (30)]. Corresponding genome assemblies for *H. contortus* (27) and *T. muris* (21) respectively, were downloaded from WormBase ParaSite (WBP) FTP server (31) and reads were mapped to these genomes using HISAT2 [v2.1.0 (32)]. Following genome mapping, raw gene counts were assigned through use of SubRead v 2.0.1 featureCounts (33). Raw counts of orthologous genes in samples were transformed to FPKM using countToFPKM (34) and median FPKMs were calculated in order to represent raw gene expressions of various developmental stages in these nematodes.

## Results and Discussion

### Parasitic Nematodes Possess *Caenorhabditis elegans* NP-GPCR Homologs

In this study we identified 1059 putative *Ce-NP-GPCR* homologs in the predicted protein datasets of 10 phylogenetically dispersed nematode parasites (see **Supplementary Table 2** and **Figure 1**). To our knowledge this is the most comprehensive analysis of *NP-GPCR* profiles in parasitic nematodes to date, spanning five phylogenetic clades and a range of parasitic lifestyles [human parasitic nematode (HPN), animal parasitic nematode (APN), plant parasitic nematode (PPN), entomopathogenic nematodes (EPN)]. Several key points emerge from this study:

#### Nematode Parasites Possess a Reduced Complement of *Caenorhabditis elegans NP-GPCR* Homologs

**Figure 2:**
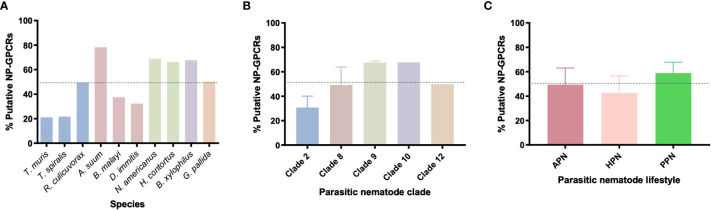
Nematode parasites have reduced and variable *NP-GPCR* complements across **(A)** 10 nematode species **(B)** phylogenetic clades and **(C)** parasitic lifestyles, expressed as a % of the predicted 152 *Caenorhabditis elegans* NP-GPCRs. Dotted line represents the average % (49.3%) of *Ce-NP-GPCRs* across all 10 species. APN, animal parasitic nematode; HPN, human parasitic nematode; PPN, plant parasitic nematode.

All 10 parasitic nematodes examined in this study exhibited restricted profiles of the 152 *Ce*-*NP-GPCRs* [21-78% *Ce*-*NP-GPCR* profile (average 49.2%); see **Supplementary Table 2**, **Supplementary Data Sheet 1** and **Figure 2**] this trend is similar to that noted previously (35, 36). *Ascaris suum* boasts the largest *Ce*-*NP-GPCR* complement of all parasites examined (78%; **Figure 2A**), including in comparison to the clade 9 species *H. contortus* and *N. americanus* which are more closely related to *C. elegans.* This suggest that *A. suum* has lost fewer NP-GPCR encoding genes than both *H. contortus* and *N. americanus* despite being more distantly related to *C. elegans.* The lowest complement of *Ce-NP-GPCRs* was identified in the clade 2 species *T. muris* (21%) and *T. spiralis* (22%; **Figure 2A**). The phylum spanning profile of NP-GPCR encoding gene complements reported here closely aligns with the parasitic nematode neuropeptide [FMRF-amide-like peptide (*flp*), and neuropeptide like protein (*nlp*)] profiles characterised previously (36, 37), where *A. suum* and the clade 2 species (*T. spiralis* and *T. muris*) also display the largest and smallest complements of parasite neuropeptide encoding genes respectively.

The HMM based approach employed also identified several biogenic amine *GPCRs* in addition to the *NP-GPCRs* reported here (data not shown); this provides confidence that all putative *NP-GPCRs* were identified in the available parasitic nematode datasets. Although a small number of divergent *NP-GPCR* sequences without an obvious *C. elegans* ortholog were identified in specific parasite species (see **Supplementary Data Sheet 1** and **Supplementary Figures 2–12**), these were not broadly conserved across the parasite species examined.

#### Nematode Parasite *NP-GPCR* Profiles Vary Within and Between Phylogenetic Clades

The NP-GPCR encoding gene profiles of parasitic nematodes representing five nematode clades [2, 8, 9, 10, 12; Holterman et al. (38)] were examined in this study. Whilst only a small number of species from each clade were examined here, all clades exhibited a reduced complement of *Ce-NP-GPCR* homologs (see **Figure 2B**); clade 9 and 10 nematodes displayed the highest complement (both 68%) of *Ce-NP-GPCR*s and clade 2 displayed the most reduced (21%).

Within clades, variation in *NP-GPCR* complement was evident; for example, whilst the clade 2 species, *T. muris* and *T. spiralis*, displayed a highly similar, reduced, *NP-GPCR* complement (21%), an additional clade 2 species, *R. culicivorax* (entomopathogenic nematode), possessed 49% of *Ce-NP-GPCR* homologs (**Figures 2A**, **B** and **Supplementary Table 2**). Similarly, the clade 8 filarids, *B. malayi* and *D. immitis*, displayed reduced *NP-GPCR* complements relative to *A. suum* (clade 8). These data suggest multiple distinct gene loss events in the lineages that led to present day *Trichuris*/*Trichinella* and filarid spp. It is also likely that some of the 152 *Ce*-*NP-GPCRs* arose from gene duplication events that occurred in the lineages that led to the crown clades (clade 8-12; Holterman et al. ()), and so the *NP-GPCRs* absent from clade 2 species may not have been present in the last common ancestor of all nematodes. In contrast, both of the plant parasitic nematodes examined, *B. xylophilus* (clade 10) and *G. pallida* (clade 12), display relatively similar *NP-GPCR* profiles despite their distinct clade designations (**Supplementary Table 2**). The number of *NP-GPCR*s present appears to be consistent across nematode lifestyles (as defined here; **Figure 2C**), however the gene profiles are different since species have distinct gene repertoires.

#### Nematode Parasite *NP-GPCR* Profiles Include Representatives of All of the Rhodopsin and Secretin NP-GPCR Families

**Figure 3:**
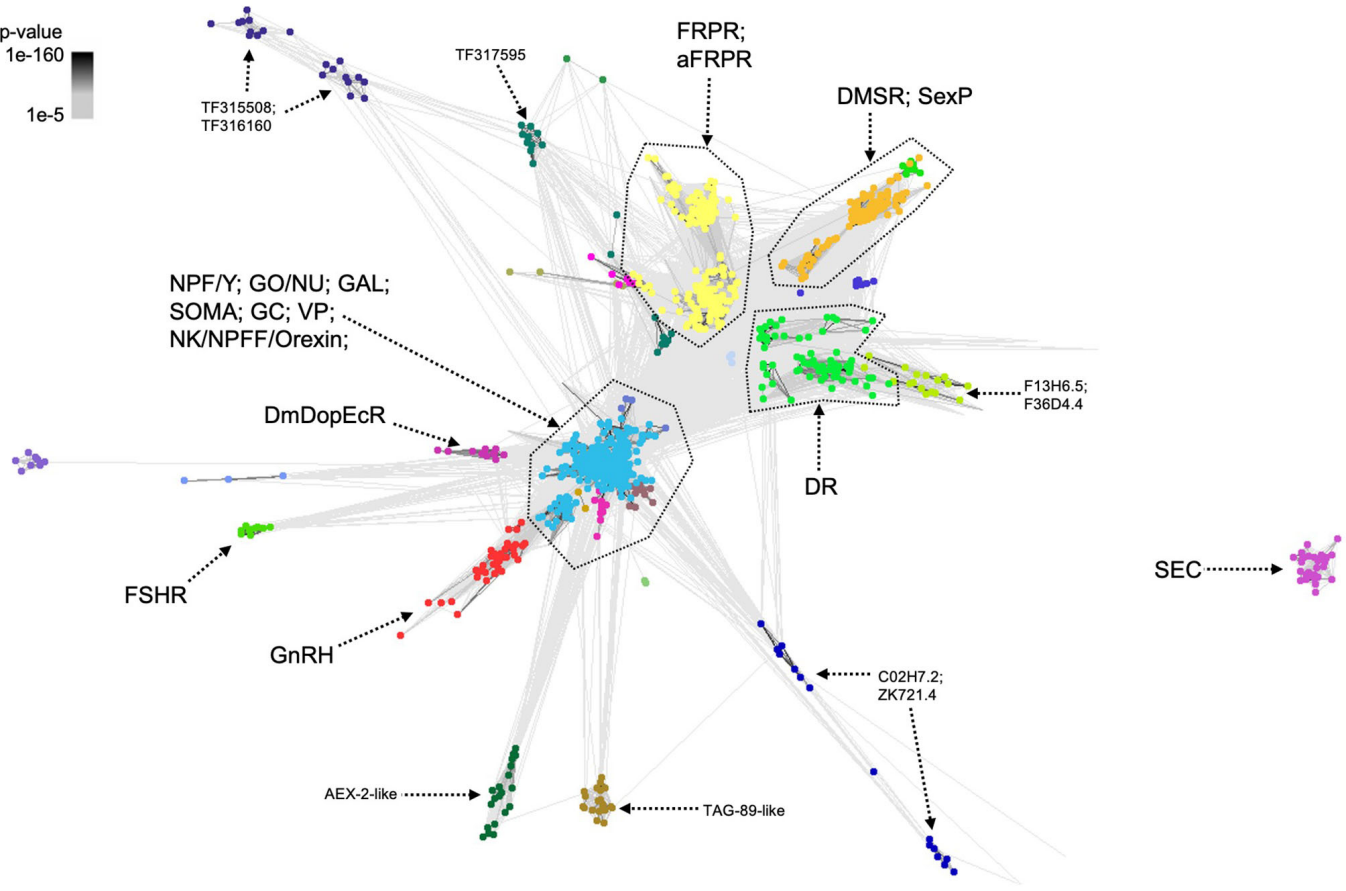
CLANS analysis identifies NP-GPCR sub-families. Similarity matrix derived from all-against-all BLASTp comparisons between all identified nematode *NP-GPCR* sequences (E-value limit = 1e-5). NPF/Y, neuropeptide F/Y receptor family; SOMA, somatostatin receptor family; GAL, galanin receptor family; FRPR, FMRFamide Peptide Receptor family; aFRPR, another FMRFamide Peptide Receptor family; DMSR, Drosophila myosuppressin receptor family; GO/NU, Ghrelin-obstatin/neuromedin U receptor family; NK/NPFF/Orexin, Neurokinin/neuropeptide FF/orexin receptor family; GnRH, Gonadotropin-releasing hormone receptor family; GC, Gastrin-cholecystokinin receptor family; VP, Related Vasopressin receptor family; SexP, Related to Sex peptide receptor family; DR, related to fly ortholog (TF315326), plus related family (TF315359); TAG-89-like, Related family with no specific orthologs (TF318526); AEX-2-like, Related family with no specific orthologs (TF316587); TF317595, Related family with fly ortholog; TF315508, Related family with no specific orthologs; TF316160, Related family with no specific orthologs; FSHR, follicle-stimulating hormone receptor; DmDopEcR, *Drosophila* Dopamine/Ecdysteroid receptor; SEC, secretin-type receptors.

The nematode parasite *NP-GPCR* profiles include representatives from the 17 rhodopsin and secretin receptor sub-families described in *C. elegans* (14). It is interesting to note that whilst there is broad representation across the majority of receptor sub-families (see **Figure 3**), there are also significant gaps in *NP-GPCR* complements especially within the *Drosophila* Dromyosuppressin (*dmsr*-10, 12-16) and *Drosophila* FMRF-amide (*frpr*-11-13) GPCR families (see **Supplementary Table 2**). Also evident are significant gaps in the otherwise broad *NP-GPCR* family profiles of the filarids including an absence of members of the Ghrelin-obstatin/neuromedin U, Galinin and Sex Peptide receptor families and a significant reduction in Neurokinin/neuropeptide FF/orexin receptor family members (see **Supplementary Table 2**).

#### Nematode Parasite *NP-GPCRs* Are Broadly Expressed but Display Differential Expression Patterns Across Nematode Life-Stage

The majority of *NP-GPCRs* are broadly expressed across the lifecycle stages of key species in this study (those with available life-stage specific RNASeq data; FPKM ≥1) indicating their general importance to nematode biology (see **Supplementary Table 3**). *NP-GPCRs* display differential expression patterns across life-stages in all parasitic nematodes examined (*T. muris*, *A. suum, B. malayi*, *D. immitis*, *H. contortus*, *G. pallida*; see **Supplementary Table 3**. Whilst it is interesting to note that the majority of *NP-GPCRs* are expressed in all life stages, including in adult nematodes, there appears to be a general upregulation of *NP-GPCR* expression in the larval stages of a number of species including *B. malayi* (L3), *T. muris* (L2), *G. pallida* (J2), *D. immitis* (microfilariae). This indicates that, whilst *NP-GPCRs* have an important role across the nematode lifecycle, there may be an enhancement of NP-GPCR signaling in the larval stages that could reflect a significant need for movement/migration and development at this stage. Whether these patterns of NP-GPCR expression can be directly tied to variation in gene function between lifecycle stages remains to be investigated.

### Nematode Parasite *In Silico NP-GPCR* Analyses Have Potential to Direct Drug Discovery Pipelines

The volume and complexity of the *NP-GPCR* profiles outlined above challenge the ability to prioritise the most attractive *NP-GPCRs* for validation as novel drug targets. *In silico* approaches offer a novel route to exploit available datasets and integrate information to direct drug target selection (39–42). Here we present a novel *in silico* nematode parasite *NP-GPCR* drug target prioritization pipeline that incorporates pan-phylum *NP-GPCR* conservation (generated in this study), parasite life-stage specific expression, and *C. elegans-*derived phenotype data to assess the target appeal of *NP-GPCRs* for nematode control (**Figure 1**).

#### *NP-GPCRs* Have Conservation Profiles That Highlight Their Appeal as Broad Spectrum Drug Targets

**Figure 4:**
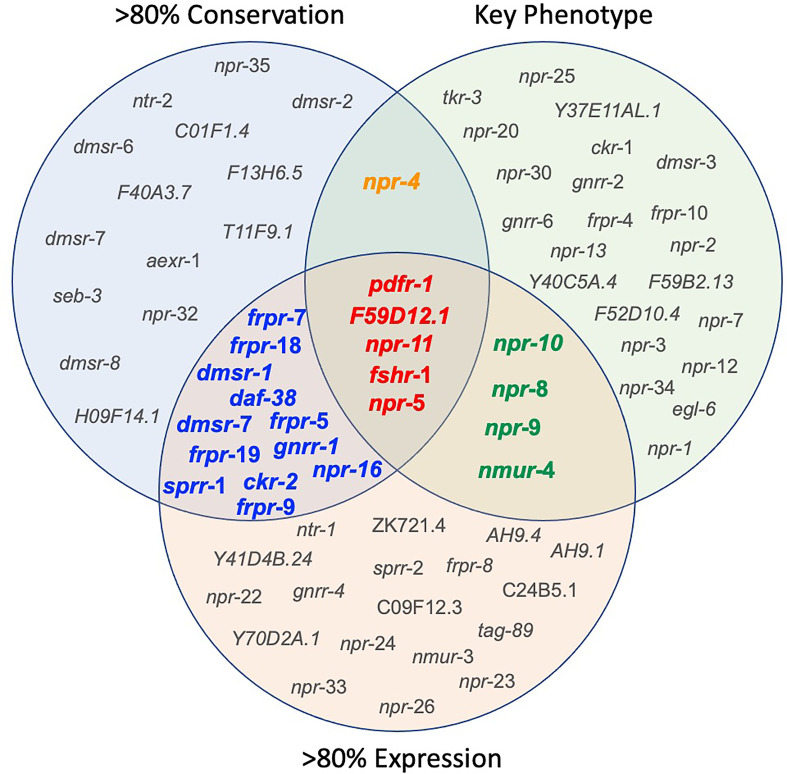
Integration of nematode ‘omics data informs *NP-GPCR* target prioritization. Venn Diagram illustrating the *NP-GPCRs* emerging from the prioritization pipeline following consideration of *NP-GPCR* conservation, *NP-GPCR* expression in key therapeutically relevant parasitic nematode lifecycle stages, and *Ce-NP-GPCR* null mutant/RNAi phenotype. Based on currently available data, the most appealing broad-spectrum *NP-GPCR* targets are highlighted in red. Receptors highlighted in orange represents those that share broad spectrum and key phenotype (lethality and locomotory) appeal; those highlighted in blue share broad spectrum conservation and expression attributes, and those highlighted in green share key phenotype and broad spectrum expression. >80% expression = *NP-GPCRs* that are expressed (FPKM>1) in more than 80% of the therapeutically relevant lifecycle stages analysed.

Seven of the 152 *Ce*-*NP-GPCRs* are conserved across all 10 parasitic nematodes examined (*gnrr*-1, *ckr*-2, *frpr*-19, *C01F1.4*, *F59D12.1*, *pdfr*-1 and *seb*-3; see **Supplementary Table 2** and **Figure 4**). An additional six *NP-GPCRs* are conserved in nine of the 10 key species examined (*npr*-4, *daf*-38, *dmsr*-2, *dmsr*-8, *T11F9.1*, *H09F14.1;* see **Supplementary Table 2** and **Figure 4**) and a further 18 are conserved in eight of the 10 parasites in this study (*npr*-5, *npr*-11, *npr*-35, *npr*-16, *npr*-32, *ntr*-2, *sprr*-1, *frpr*-5, *frpr*-7, *frpr*-9, *frpr*-18, *dmsr*-1, *dmsr*-6, *dmsr*-7, *F40A3.7*, *aexr*-1, *fshr*-1, *F13H6.5;* see **Supplementary Table 2** and **Figure 4**). Eighteen *NP-GPCRs* were not identified in any parasite species (see **Supplementary Table 2**), and six *NP-GPCRs* (*dmsr-*11, *frpr*-16, *gnrr*-7, *npr*-33, *D1014.2*, *ZK863.1*; present in only one species) show highly restricted patterns of conservation.

#### *NP-GPCRs* Are Associated With *C. elegans* Phenotypes That May Have Drug Target Appeal

*Caenorhabditis elegans* functional data may inform *NP-GPCR* target appeal through the collation and consideration of phenotype information. In this study we collated phenotype data from *C. elegans* null mutant/RNAi experiments for the 152 *NP-GPCRs* [see WormBase (15); see **Supplementary Table 4**]. Each *NP-GPCR* was scored based on perceived phenotype significance to nematode biology and/or established anthelmintic endpoints, and therefore potential drug target appeal (see ***Materials and Methods***). Often, multiple phenotypes were attributed to individual *NP-GPCRs*, therefore scores were added to yield an overall phenotype score for each receptor (see **Supplementary Table 4**). Several key points emerge from these datasets: (i) 89 of the 152 putative *Ce*-*NP-GPCRs* had no associated null mutant/RNAi phenotype(s) which may reflect a combination of: (a) lack of functional analyses data for *Ce-NP-GPCRs*, (b) use of an unsuitable *C. elegans* post-functional genomics bioassay and/or, (c) functional redundancy in nematode neuropeptidergic signalling systems; (ii) 16 *Ce-NP-GPCRs* had a lethal phenotype reported in at least one study (*npr*-5, *nmur*-4, *npr-*20, *tkr-*3, *npr-*30, *gnrr*-2, *gnrr-*6, *ckr-*1, *frpr-*4, *frpr*-10, *dmsr*-3, *Y37E11AL.1*, *Y40C5A.4, F59B2.13, F52D10.4, fshr*-1; see **Supplementary Table 4**); (iii) *npr*-3, -4, -7, *-*11, *-*12, -34, *egl*-6 and *pdfr-*1 also scored highly as these *NP-GPCRs* are associated with atypical locomotion, sterility or reproductive phenotypes in at least one study. Although the scoring system adopted here elevates the scores of *NP-GPCRs* that fall into multiple phenotype categories, the appeal of mutant/RNAi phenotypes associated with, for example, only locomotion should not be ignored; the *NP-GPCRs* associated with locomotion (in at least one study) include *npr*-1, -2, -8, -9, -10, -13, -25, and F59D12.1.

It is interesting to note that of the 16 *NP-GPCRs* that are associated with lethal phenotypes in *C. elegans* three (*npr-*5, *ckr-*1 and *Y40C5A.4*) are present in the most important APN/HPN species in this study (*A. suum*, *B. malayi*, *D. immitis*, *N. americanus*, *H. contortus*; see **Supplementary Table 2**). 16 additional *NP-GPCRs* emerged from the available phenotype data with appealing locomotory, reproductive and/or sterility phenotypes. Of these, five (*npr-1, -*11, -13, *pdfr*-1, *F59D12.1*) are present in the most important APN/HPN species in this study (see **Supplementary Table 2**). Finally, of the seven *NP-GPCRs* completely conserved in the parasitic species examined in this study, *F59D12.1* and *pdfr-1* have been linked to deleterious *C. elegans* phenotype post RNAi/knockout (see **Supplementary Table 4**).

In the context of this study, there are several important caveats to the extrapolation of the WormBase derived *C. elegans* phenotype data for drug target prioritisation including: (i) the reported differences between phenotypes recorded for multiple distinct mutations associated with the same gene, as well as RNAi animals (typically performed in RNAi hypersensitive mutant strains), (ii) the variable and often specific nature of the phenotype screens employed, and (iii) the bias in the volume/quality of functional data for specific NP-GPCRs or GPCR families. Whilst we have attempted to incorporate all of the observed *C. elegans* phenotypes recorded on WormBase regardless of experimental approach (how the mutant was generated, phenotype screens employed), the major caveats outlined above somewhat limit the utility of these data, and emphasise the need for functional analysis of all highly conserved and highly expressed NP-GPCRs in parasitic nematodes. Despite this, the approach offers a route to prioritising drug target candidates for functional validation in low throughput parasite platforms.

The format of our prioritisation pipeline allows for the distinct prioritisation of NP-GPCRs based on conservation, expression and/or *C. elegans* phenotype. This enables for the segregation or integration of prioritisation criteria as required and for the addition of phenotype data as they become available.

#### *NP-GPCRs* Are Broadly Expressed Across Nematode Life-Stages Underpinning Their Appeal as Novel Drug Targets

The available nematode RNASeq data suggest that parasite *NP-GPCRs* are broadly expressed across the species examined in this study (see **Supplementary Table 3**; for example, of the 13 genes that were conserved in at least nine of the 10 parasite species examined (see *NP-GPCRs Have Conservation Profiles That Highlight Their Appeal as Broad Spectrum Drug Targets*), the majority are also expressed in therapeutically relevant lifecycle stages (including: adult *H. contortus*; microfilariae and adult *B. malayi* and *D. immitis*; adult *A. suum*; adult *T. muris*; see **Supplementary Table 3**) underpinning the appeal of *NP-GPCRs* as therapeutic targets. In this context, the *NP-GPCR* expression data on their own do not discriminate sufficiently to prioritise a reduced cohort of broad spectrum drug targets however, in the scenario where a species focused/narrow spectrum target is desirable, ranking candidate drug targets based on expression data is more informative. For example, 21 *NP-GPCRs* are expressed in all therapeutically relevant stages of *A. suum* (*npr-*1, -16, -23, -33*, gnrr-*2, *daf-*38*, ckr-*2*, frpr-*5, -7, -9, -18, -19, *sprr-*1*, dmsr-*1, -4*, C17H11.1, C24B5.1, tag-*89*, fshr-*1*, F59D12.1*, *pdfr-*1); of these, *npr*-1, *fshr*-1, *F59D12.1* and *pdfr-*1 also display defective phenotypes in *C. elegans* (see *NP-GPCRs Are Associated With C. elegans Phenotypes That May Have Drug Target Appeal*).

#### Several Parasitic Nematode *NP-GPCRs* Emerge as the Most Appealing Broad Spectrum Drug Targets

The data presented here identify 17 *NP-GPCRs* as the most appealing broad spectrum drug target candidates (>80% conservation and expression across key parasitic nematodes; see **Figures 1** and **4**). Parasitic nematode reverse genetics platforms are low throughput necessitating a focus on a smaller subset of *NP-GPCRs*. With this in mind, *npr-5*, *npr-11*, *pdfr-1*, *fshr-1* and F59D12.1 step forward as initial candidates for functional validation (**Figures 1** and **4**).

Two of the *NP-GPCRs* that emerge from our pipeline as appealing targets *(npr*-5 and *-*11*)* have been linked to several peptides. NPR-11 has been functionally linked to NLP-1 and FLP-34 and, heterologously matched with FLP-21, -18, -34, -15 and -27 (43–47). NPR-5 is also functionally linked to FLP-18 and heterologously linked to FLP-18 and -21 (46, 48–50). Interestingly FLP-18 signalling has been shown to be important to nematode biology and is associated with a raft of biological processes including chemosensation, heat avoidance, reversal length, foraging behaviour, metabolism, locomotion quiescence during lethargus, and dauer formation (49–55); these data enhance the appeal of NPR-5 and -11 as putative novel drug targets. Significantly, *npr*-5 is conserved in 84% of 134 nematode genomes, representing 109 species, 7 clades and 3 distinct lifestyles (56), highlighting the importance of NPR-5 across phylum Nematoda.

PDFR-1 is related to arthropod Pigment Dispersing Factor Receptor and, more distantly, to vertebrate Calcitonin and Vasoactive Intestinal Peptide receptors (57). These receptors function in the control of circadian rhythms and arousal (51, 57, 58). PDFR-1 in *C. elegans* has been deorphanised heterologously and functionally to PDF-1 and PDF-2 (NLP-37) peptides, which modulate locomotion (57, 59). PDFR-1 signalling has also been implicated in inducing extended roaming states, arousal of locomotory behaviour following lethargus, and in the promotion of male mate searching behaviour in *C. elegans* (51, 60, 61). Notably, *pdf*-1 and *pdfr*-1 were present together in 96% of 134 nematode genomes (unpublished observations).

The remaining prioritised receptors are orphan NP-GPCRs that have not yet been linked to a cognate ligand. F59D12.1, also known as PCDR-1 (Pathogen Clearance Defective Receptor), has been associated with locomotion *via* RNAi experiments which resulted in slow and paralyzed worms (62). PCDR-1 also plays a key role in pathogen clearance of *Microbacterium nematophilum* infection in *C. elegans* (63). The functional data available for *fshr-*1 indicate that mutant *C. elegans* (tm3954) and RNAi worms display lethal phenotypes (15, 64).

**Figure 5:**
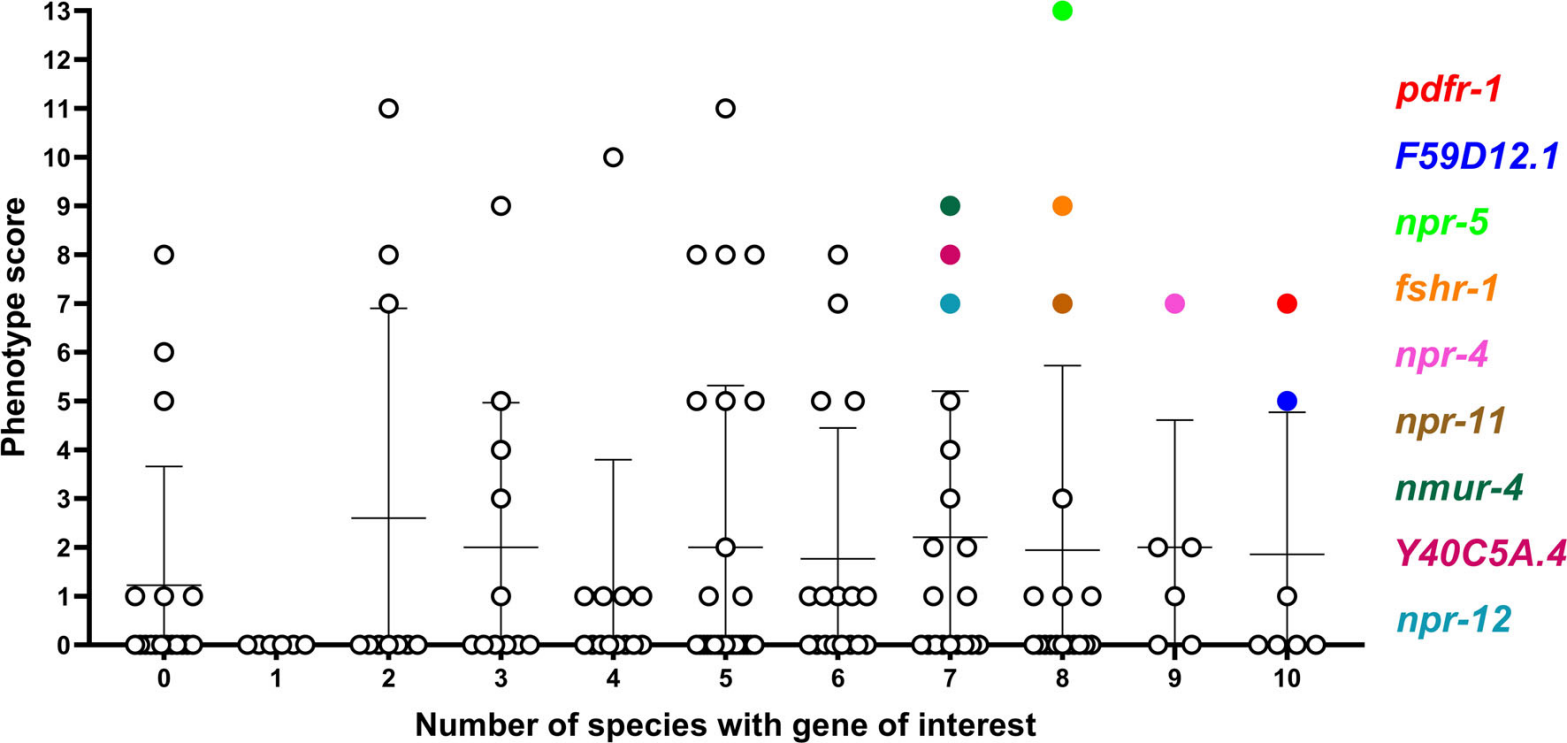
Comparison of phenotype score and *NP-GPCR* conservation. Phenotype score (sum of all phenotype categories associated with each gene) was plotted against the number of species which possess an *NP-GPCR* of interest. There is a no statistically significant correlation (Spearman rho) between phenotype score and NP-GPCR encoding gene conservation. Only genes displaying both a high level of conservation and phenotype score are highlighted.

Whilst several additional *NP-GPCRs* (*gnrr*-1, *ckr*-2, *frpr*-19, *C01F1.4*, *seb*-3) were conserved in all species examined in this study, the limited functional information available for these receptors has precluded their prioritisation as the most appealing targets at this point (see **Figure 4**). This reflects a major gap in the *NP-GPCR* null mutant/RNAi phenotype data and is a caveat to drug prioritisation in this context. In addition, the scale and scope of the post-functional genomics phenotype screens performed in *Ce-NP-GPCR* null mutant/RNAi experiments are: (i) highly variable and often gene dependent and, (ii) focus almost exclusively on loss of function screens (lack of over-expression data), such that this results in a degree of bias within the *NP-GPCR* prioritisation pipeline whereby highly conserved receptors that simply lack phenotype data are not emerging among the prioritised subset. Indeed, this is supported by a lack of correlation between parasitic nematode *NP-GPCR* conservation and the *C. elegans* derived phenotype data reported here (Spearman’s rho; **Figure 5**). It is also interesting to note that some of the *NP-GPCRs* highlighted above, that are broadly conserved but were not prioritised due to lack of phenotype data, are also broadly expressed across nematode lifecycle stages (e.g. *gnrr*-1, *ckr*-2, *frpr-*19 expressed in 83%, 97% and 100% of the transcriptomes examined respectively); this suggests potential functional importance and should form the focus of future functional analyses in parasitic nematodes.

#### *NP-GPCR* Prioritisation Pipelines Can Also Direct Narrow Spectrum Drug Target Prioritisation

Narrow spectrum anthelmintics have proven utility in nematode parasite control strategies (65). The drug target prioritisation pipeline presented here also enables the prioritisation of narrow spectrum drug targets that are relevant to the control of specific parasite species or life-stages. For example, no macrofilaricidal anthelmintics currently exist (66). Several *NP-GPCRs* (*npr-*5, -19, -23, -29, *gnrr*-1, -4, *ckr*-1, -2, *frpr*-8, -19, *Y40C5A.4*, *aexr*-1, *fshr*-1 and F59D12.1) emerge from our pipeline as *NP-GPCRs* that are expressed in both adult male and female *B. malayi* and *D. immitis* (see **Supplementary Table 3**). Some of these NP-GPCRs have already been prioritised as broad-spectrum targets (see *Several Parasitic Nematode NP-GPCRs Emerge as the Most Appealing Broad Spectrum Drug Targets*; *npr-*5, *fshr*-1 and F59D12.1) however, an additional two *NP-GPCRs* (*ckr*-1 and *Y40C5A.4*) emerge that are also appealing as microfilaricides; this underscores the utility of the *NP-GPCR* prioritisation pipeline in teasing out species specific therapeutic targets (see **Supplementary Tables 2**–**4**).

## Conclusions

Recently improved parasite ‘omics’ data have driven a paradigm-shift towards mechanism-directed drug target screening approaches, providing an opportunity to identify the most attractive nematode parasite targets. Our focus on NP-GPCRs as therapeutic targets is driven by their importance to nematode biology (8), however the number and diversity of nematode NP-GPCRs is currently a hinderance to functional validation and successful exploitation. Here we present data on *NP-GPCR* conservation and the application of a drug target prioritisation pipeline that highlights the most attractive parasitic nematode *NP-GPCRs* for parasite control at this time. These data: (i) provide a comprehensive library of *NP-GPCRs* in key nematode parasites; (ii) enable the selection of both broad and narrow spectrum control targets; (iii) inform future validation efforts for *NP-GPCRs* in key parasitic nematode systems which are currently significantly lacking and, (iv) will expedite the anthelmintic development pipeline *via* informed target selection.

## Data Availability Statement

The original contributions presented in the study are included in the article/**Supplementary Material**. Further inquiries can be directed to the corresponding author.

## Author Contributions

AM, LA, CM, AGM, and NM designed the research. LA, CM, BC, PM, DM, AI, FM, and BAR performed the research. LA, CM, and BC analysed the data with assistance from BAR and MM. AM, LA, CM, AGM, NM, and JH wrote the manuscript. All authors contributed to the article and approved the submitted version.

## Funding

This work was supported by: the Biotechnology and Biological Sciences Research Council (BB/H019472/1 to AM); the Biotechnology and Biological Sciences Research Council/Merial Animal Health grant (BB/M010392/1 to AM, NM, and AGM); the Biotechnology and Biological Sciences Research Council/Boehringer Ingelheim (BB/T016396/1 to AM, NM, AGM, and LA); the Department of Education and Learning for Northern Ireland (studentships awarded to FM and BC); the Department of Agriculture, Environment and Rural Affairs for Northern Ireland (studentships awarded to DM and AI); the National Institutes of Health-National Institute of Allergy and Infectious Diseases grant (AI159450 to MM).

## Conflict of Interest

The authors declare that the research was conducted in the absence of any commercial or financial relationships that could be construed as a potential conflict of interest.

## Publisher’s Note

All claims expressed in this article are solely those of the authors and do not necessarily represent those of their affiliated organizations, or those of the publisher, the editors and the reviewers. Any product that may be evaluated in this article, or claim that may be made by its manufacturer, is not guaranteed or endorsed by the publisher.

## Supplementary Material

Supplementary Figure 1 Nematode parasite NP-GPCR predicted transmembrane domains. Sequences with < 4 transmembrane (TM) domains were excluded from further phylogenetic analysis. Full length sequences are expected to contain 7 TM domains. Proportion of GPCRs presenting 7 TM domains can be used as a crude but relevant proxy for NP-GPCR annotation/dataset quality in this instance.

Supplementary Figure 2–12 Maximum likelihood phylogenies constructed using the LG model of evolution (+*G* +*I*; with 5 discrete Gamma categories), 500 bootstrap replicates, partial deletion of gaps (80% site coverage cut-off) and the nearest-neighbour interchange algorithm with no branch swap filter. For rhodopsin-type GPCR phylogenies, trees were rooted using a selection of *C. elegans* biogenic amine receptors.

Supplementary Data Sheet 1 HMM search output and post-phylogenetic analysis summary highlighting the specific nematode parasite NP-GPCR encoding gene IDs identified here.

Supplementary Table 1 Parasitic nematode species examined in this study. APN, animal parasitic nematode; HPN, human parasitic nematode; PPN, plant parasitic nematode; EPN, entomopathogenic nematode. Genomes and transcriptomes used: *Trichuris muris* (21), *Trichinella spiralis* (67), *Romanomermis culicivorax* (68), *Ascaris suum* (22, 23)*, Brugia malayi* (24, 25, 69)*, Dirofilaria immitis* (26, 70)*, Necator americanus* (71)*, Haemonchus contortus* (27), *Bursaphelenchus xylophilus* (72), *Globodera pallida* (28).

Supplementary Table 2 Ce-NP-GPCR complements of 10 key nematode species. A black box indicates the presence of a homologue identified via BLAST search. A grey box indicates the absence of a gene. GO/NU, Ghrelin-obstatin/neuromedin U receptor family; NK/NPFF/Orexin, Neurokinin/neuropeptide FF/orexin receptor family; GnRH, Gonadotropin-releasing hormone receptor family; GC, Gastrin-cholecystokinin receptor family; VP, Related Vasopressin receptor family; Sex P, Related to Sex peptide receptor family; Drosophila related, related to fly ortholog (CG33639); FSHR, follicle-stimulating hormone receptor; DmDopEcR, Drosophila Dopamine/Ecdysteroid receptor.

Supplementary Table 3 Ce-NP-GPCR binary expression patterns in parasitic nematodes. A black box indicates the presence of a NP-GPCR homologue that is not expressed in a specific life cycle stage (FPKM <1). A red box indicates the presence of a NP-GPCR homologue expressed in a specific life cycle stage (FPKM > 1). A grey box indicates the absence of a gene. GO/NU, Ghrelin-obstatin/neuromedin U receptor family; NK/NPFF/Orexin, Neurokinin/neuropeptide FF/orexin receptor family; GnRH, Gonadotropin-releasing hormone receptor family; GC, Gastrin-cholecystokinin receptor family; VP, Related Vasopressin receptor family; SexP, Related to Sex peptide receptor family; Drosophila related, related to fly ortholog (CG33639); FSHR, follicle-stimulating hormone receptor; DmDopEcR, Drosophila Dopamine/Ecdysteroid receptor.

Supplementary Table 4 Ce-NP-GPCR knock out/RNAi phenotype summary. A coloured box indicates phenotype linked to each Ce-NP-GPCR encoding gene. A grey box indicates the absence of a phenotype category. GO/NU, Ghrelin-obstatin/neuromedin U receptor family; NK/NPFF/Orexin, Neurokinin/neuropeptide FF/orexin receptor family; GnRH, Gonadotropin-releasing hormone receptor family; GC, Gastrin-cholecystokinin receptor family; VP, Related Vasopressin receptor family; SexP, Related to Sex peptide receptor family; Drosophila related, related to fly ortholog (CG33639); FSHR, follicle-stimulating hormone receptor; DmDopEcR, Drosophila Dopamine/Ecdysteroid receptor.
