# Supplementary figures and images for: Phylum-Spanning Neuropeptide GPCR Identification and Prioritization: Shaping Drug Target Discovery Pipelines for Nematode Parasite Control
Source: Front Endocrinol (Lausanne). 2021 Sep 30;12:718363. doi: 10.3389/fendo.2021.718363 (PMC8515059; doi:10.3389/fendo.2021.718363)

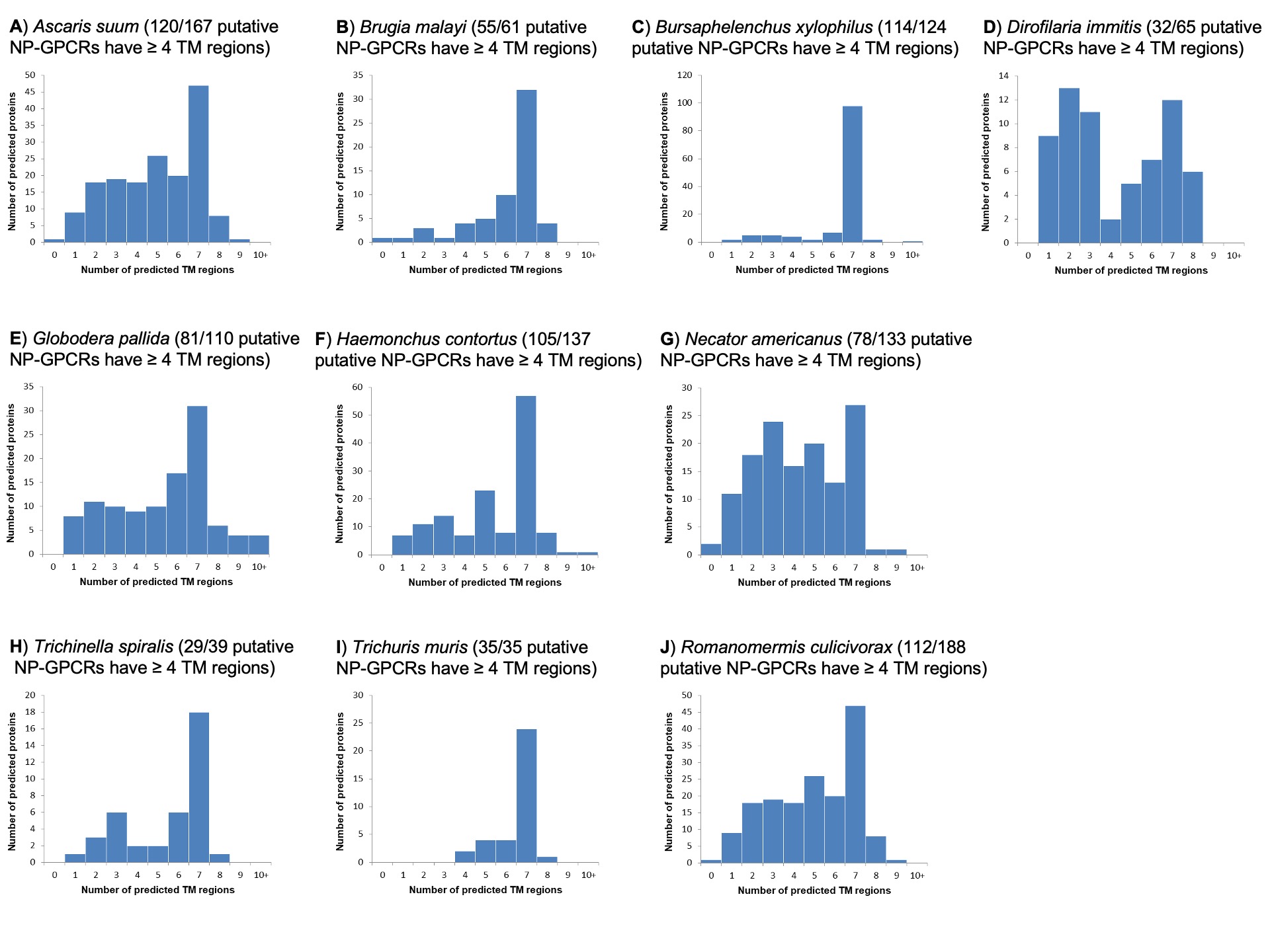

Supplement: Supplementary Figure 1 — Nematode parasite NP-GPCR predicted transmembrane domains. Sequences with < 4 transmembrane (TM) domains were excluded from further phylogenetic analysis. Full length sequences are expected to contain 7 TM domains. Proportion of GPCRs presenting 7 TM domains can be used as a crude but relevant proxy for NP-GPCR annotation/dataset quality in this instance. [file Image_1.jpg]

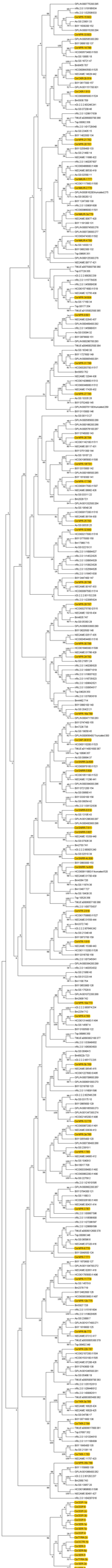

Supplement: Supplementary Figure 2–12 — Maximum likelihood phylogenies constructed using the LG model of evolution (+G +I; with 5 discrete Gamma categories), 500 bootstrap replicates, partial deletion of gaps (80% site coverage cut-off) and the nearest-neighbour interchange algorithm with no branch swap filter. For rhodopsin-type GPCR phylogenies, trees were rooted using a selection of C. elegans biogenic amine receptors. [file Image_2.jpeg]

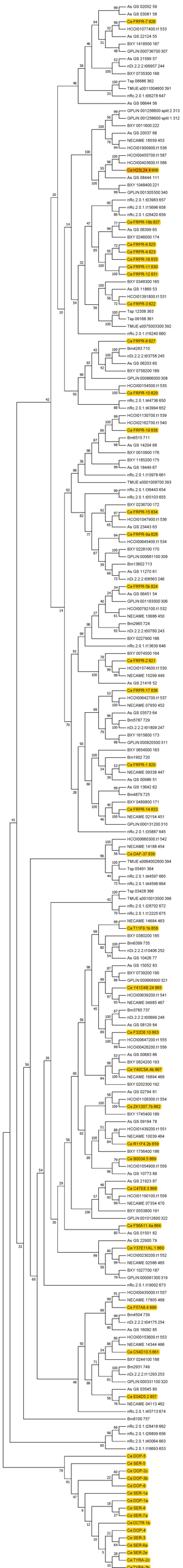

Supplement: Supplementary file 3 [file Image_3.jpeg]

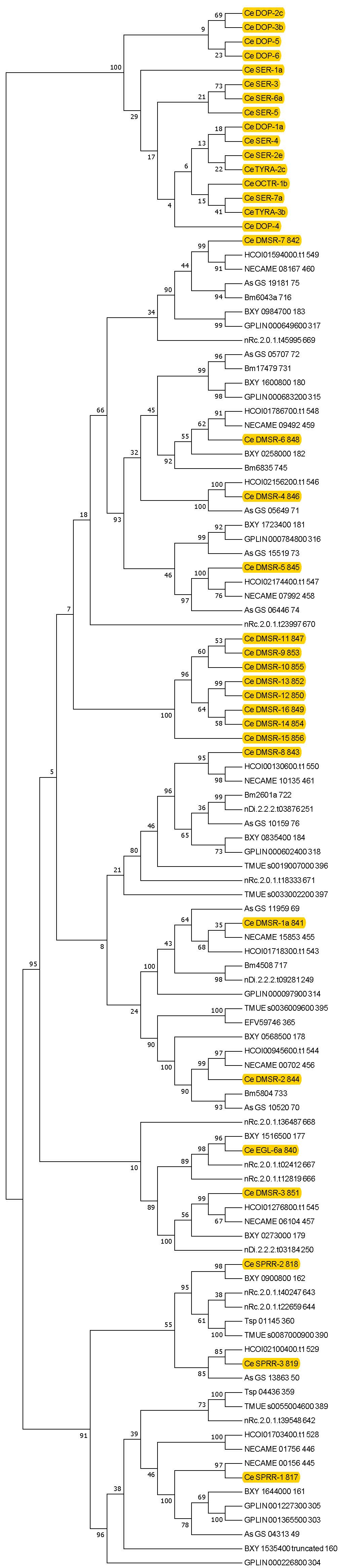

Supplement: Supplementary file 4 [file Image_4.jpeg]

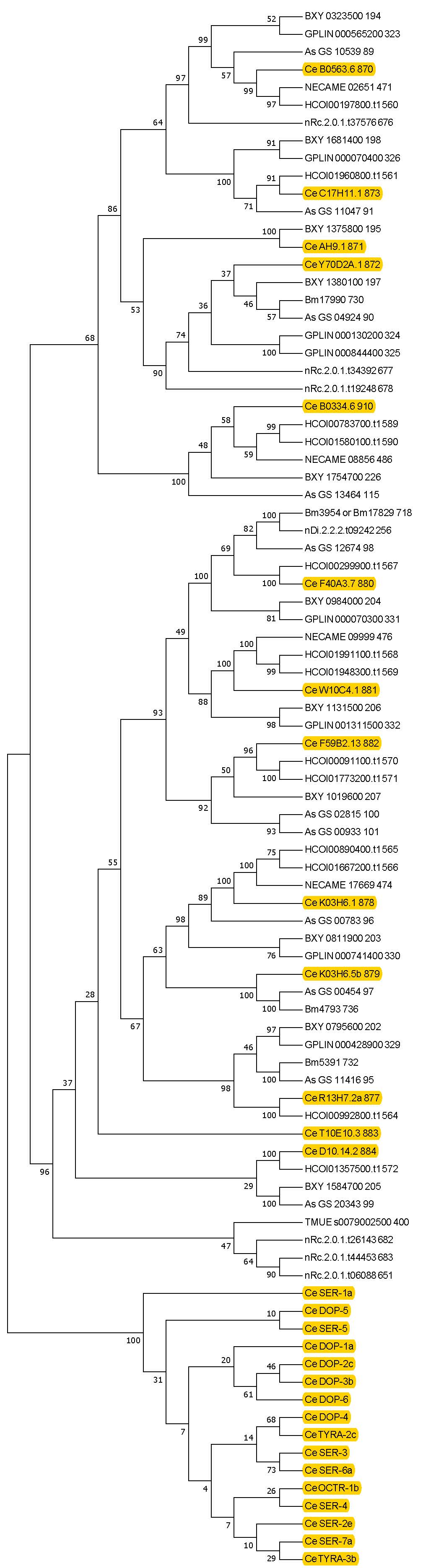

Supplement: Supplementary file 5 [file Image_5.jpeg]

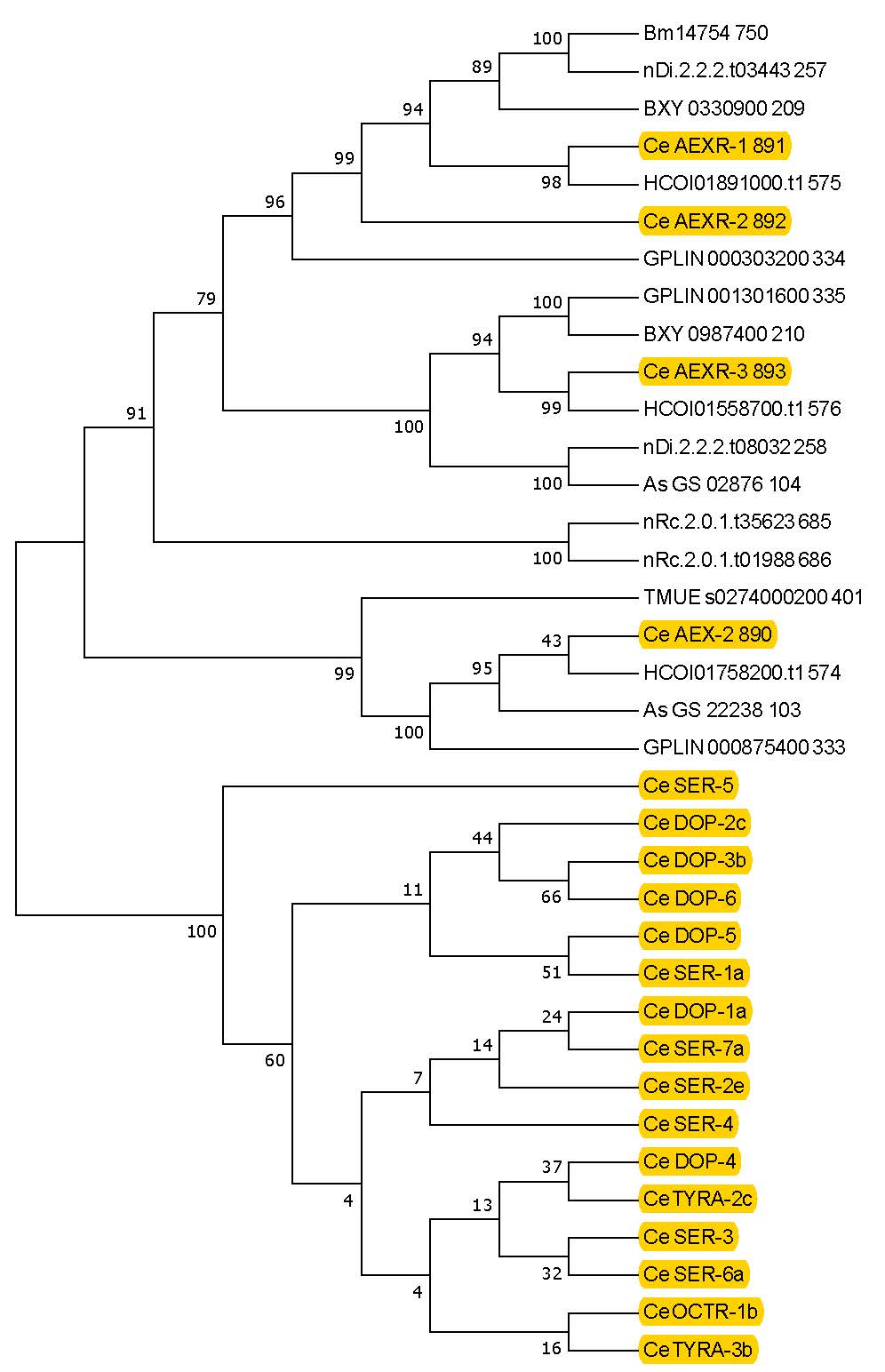

Supplement: Supplementary file 6 [file Image_6.jpeg]

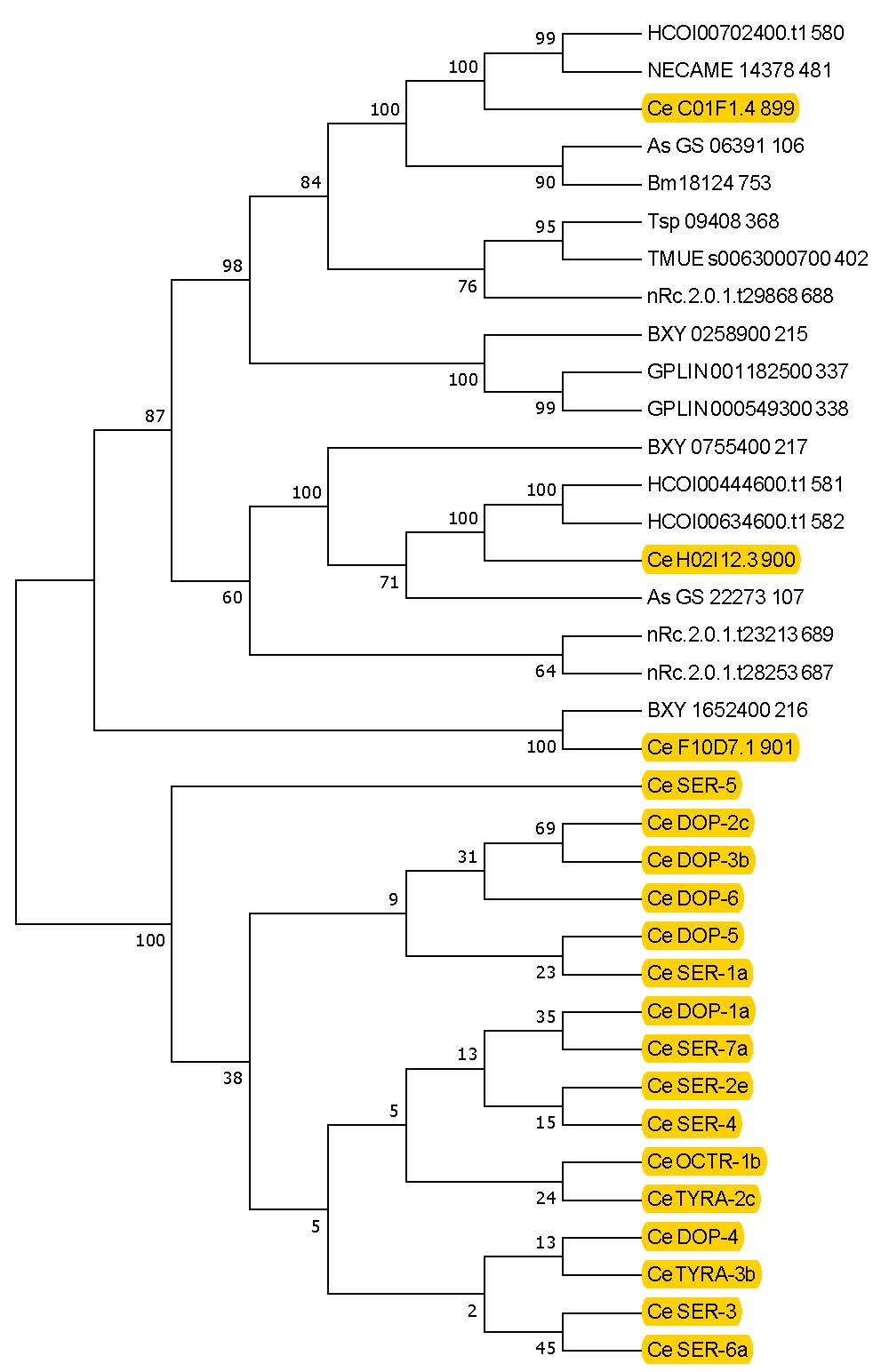

Supplement: Supplementary file 7 [file Image_7.jpeg]

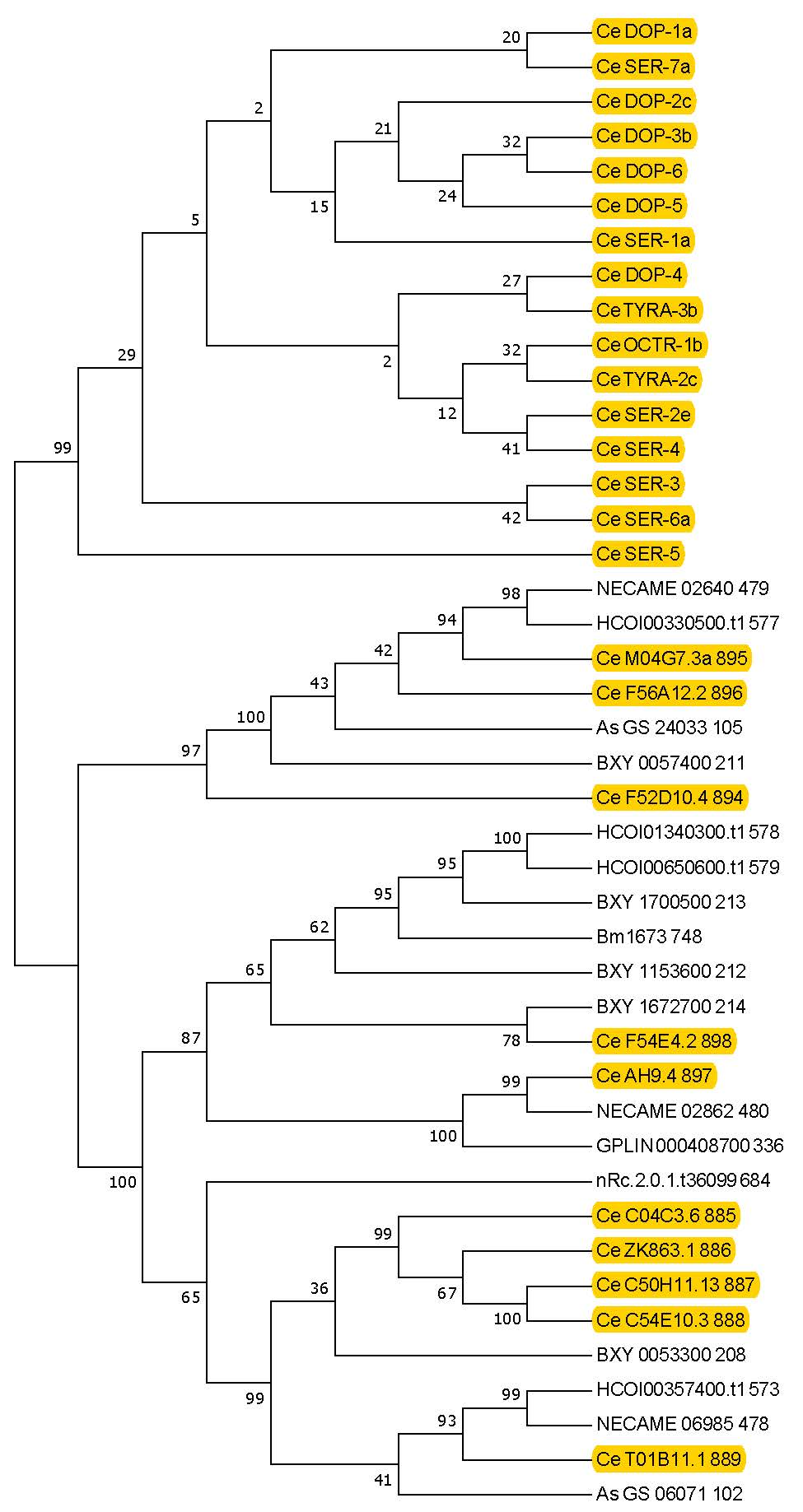

Supplement: Supplementary file 8 [file Image_8.jpeg]

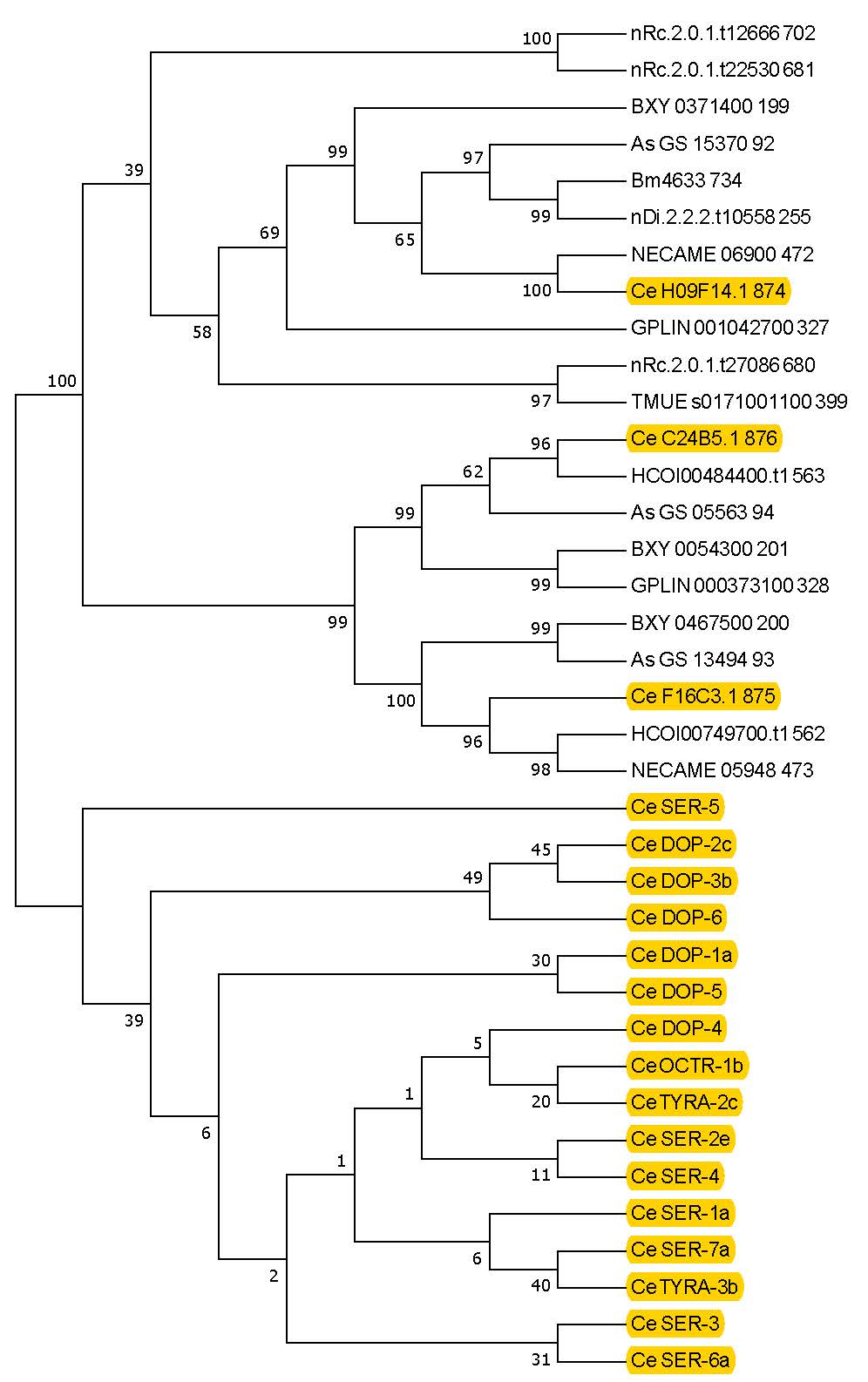

Supplement: Supplementary file 9 [file Image_9.jpeg]

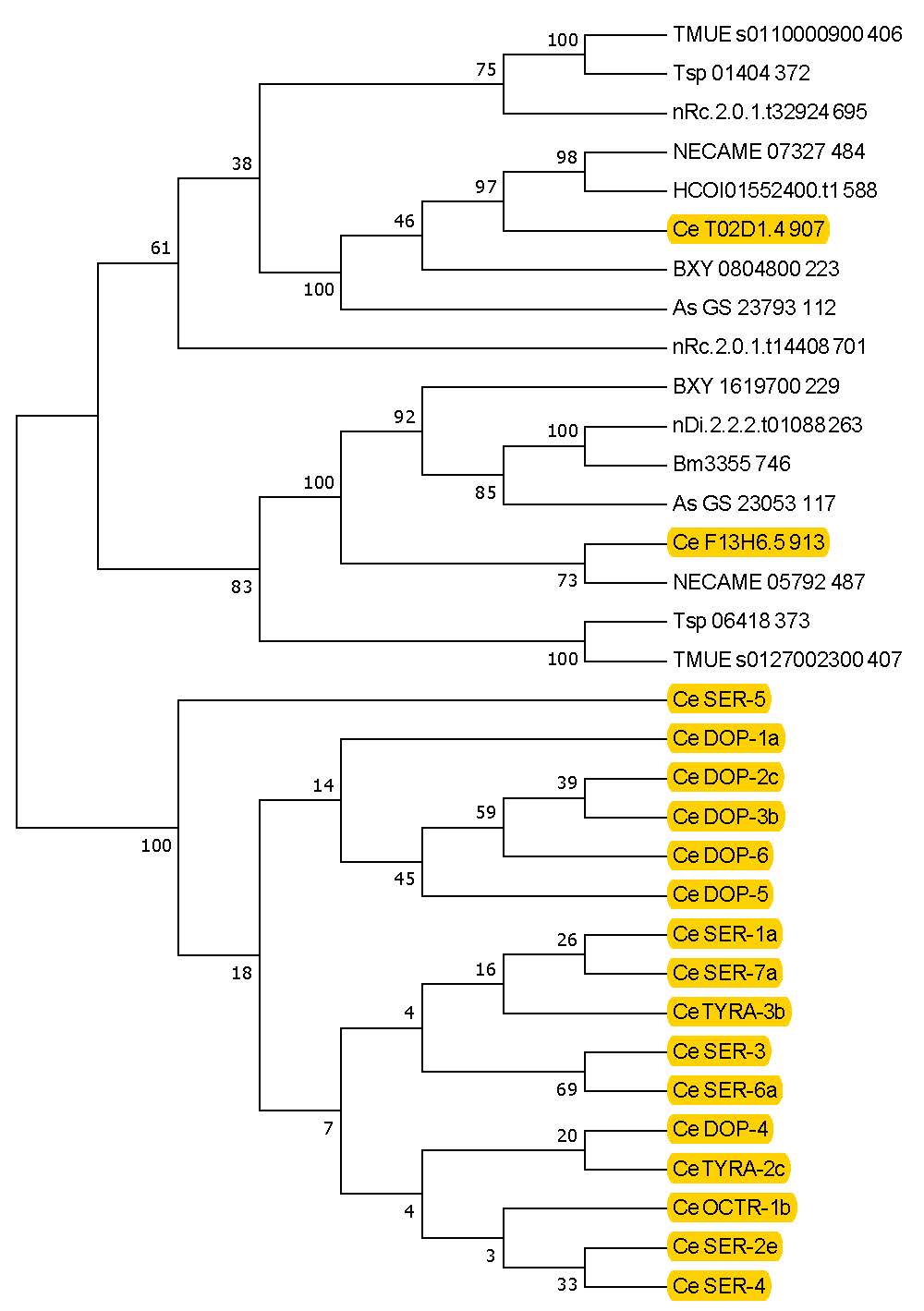

Supplement: Supplementary file 10 [file Image_10.jpeg]

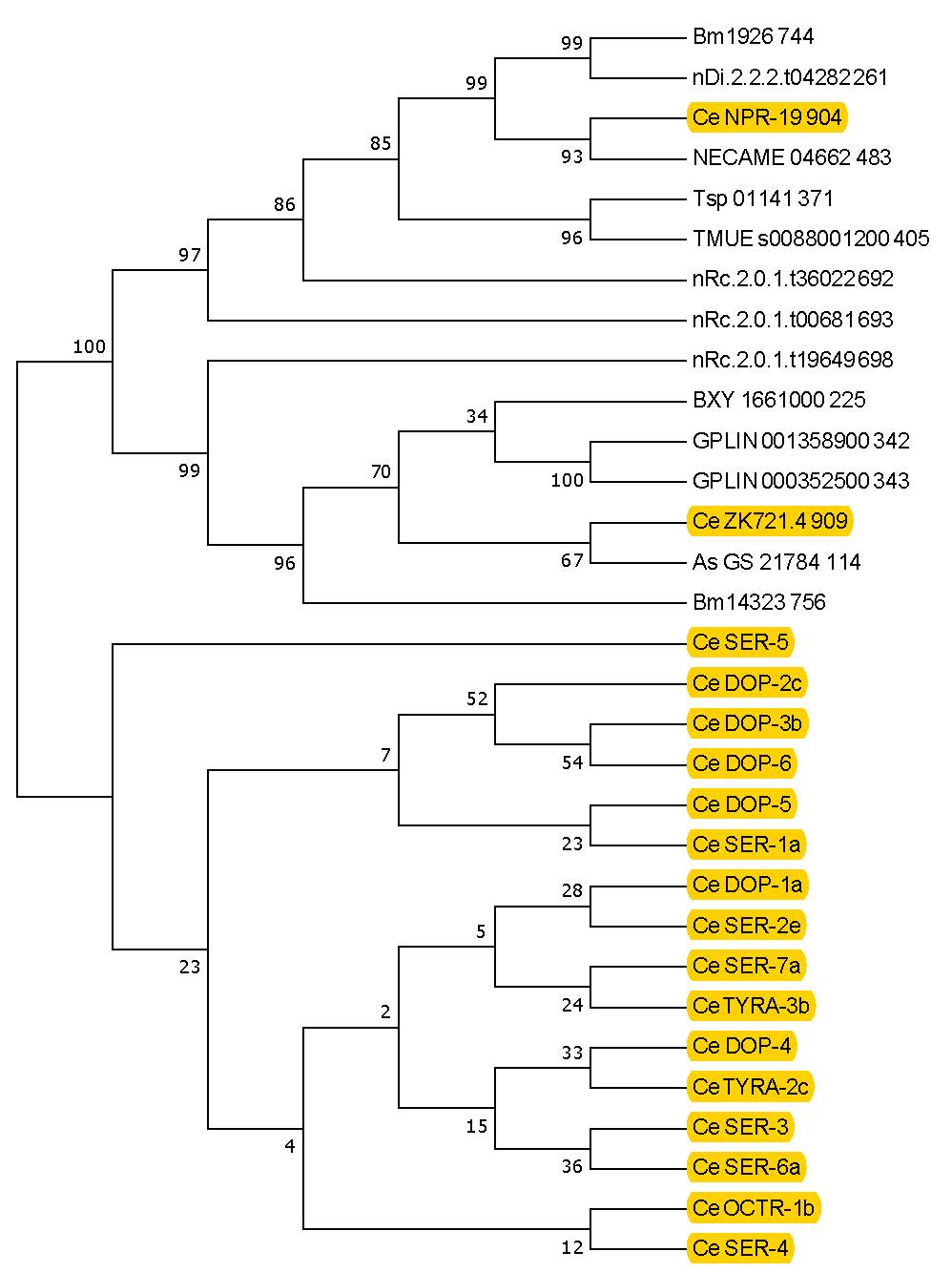

Supplement: Supplementary file 11 [file Image_11.jpeg]

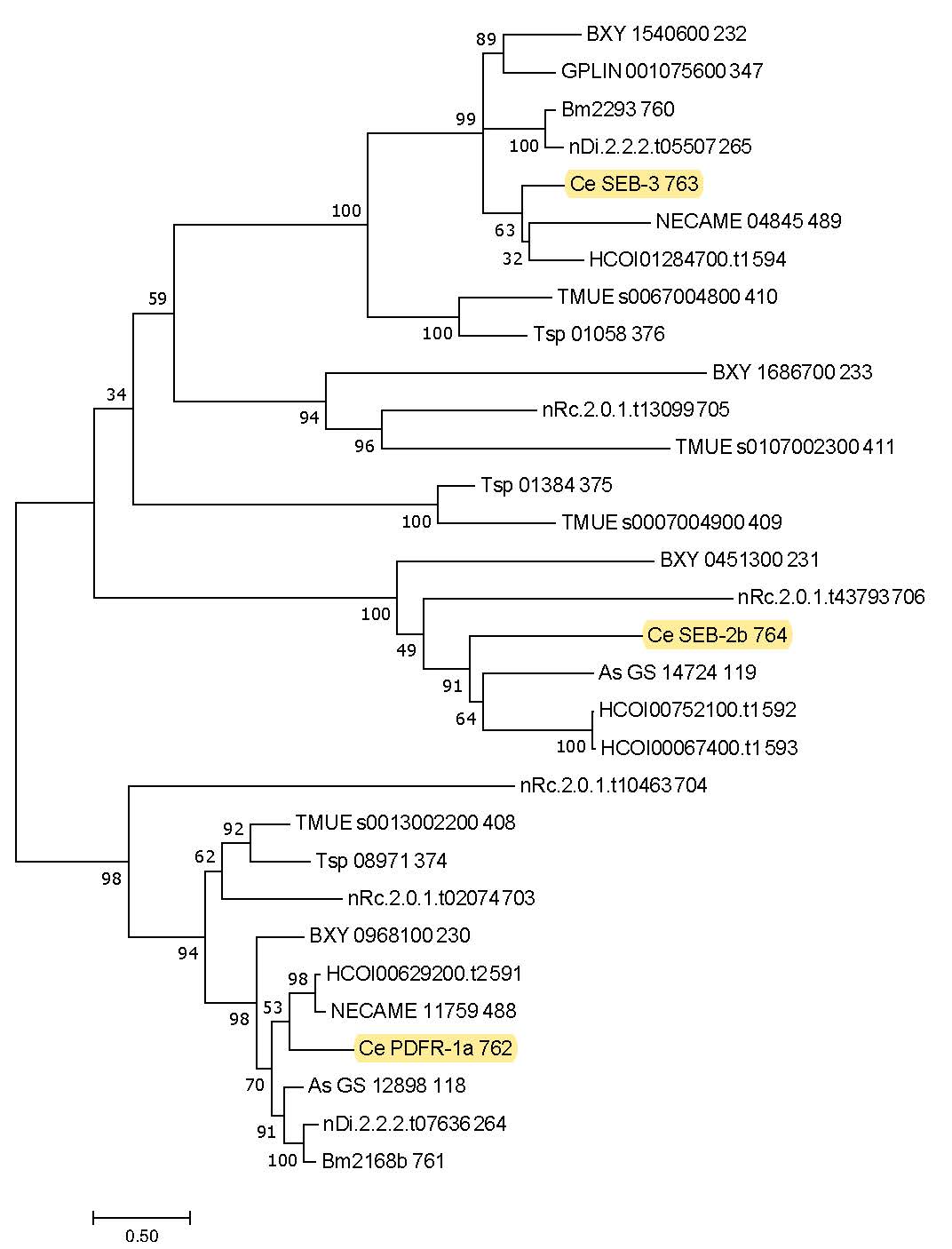

Supplement: Supplementary file 12 [file Image_12.jpeg]
