# Supplementary material for: Phylum-Spanning Neuropeptide GPCR Identification and Prioritization: Shaping Drug Target Discovery Pipelines for Nematode Parasite Control
Source: Front Endocrinol (Lausanne). 2021 Sep 30;12:718363. doi: 10.3389/fendo.2021.718363 (PMC8515059; doi:10.3389/fendo.2021.718363)
Supplement: Supplementary Data Sheet 1 — HMM search output and post-phylogenetic analysis summary highlighting the specific nematode parasite NP-GPCR encoding gene IDs identified here. [file DataSheet_1.pdf]

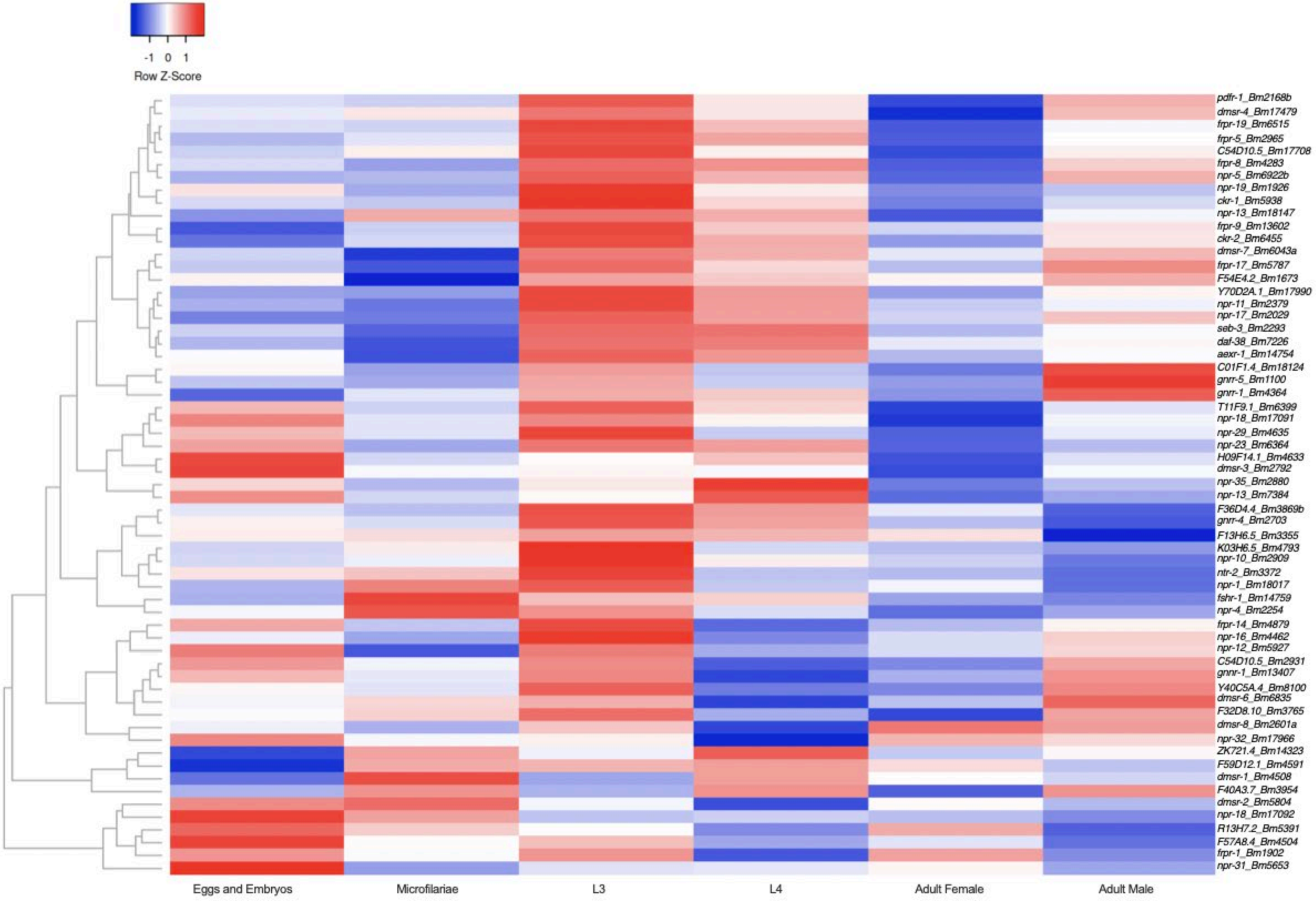

Expression heatmap generated from log2TPM values of 61 genes in *Brugia malayi* identified from developmentally staged RNA-seq libraries and https://parasite.wormbase.org utilising Heatmapper's Expression program [http://heatmapper.ca/expression, (Babicki et al., 2016)]. Average Clustering Method & Pearson's Distance Measurement Method were used. Life stages are represented in columns (Eggs and Embryos, Microfilariae, L3, L4, Adult Female, Adult Male). Rows indicated individual genes, denoted by the gene ID.

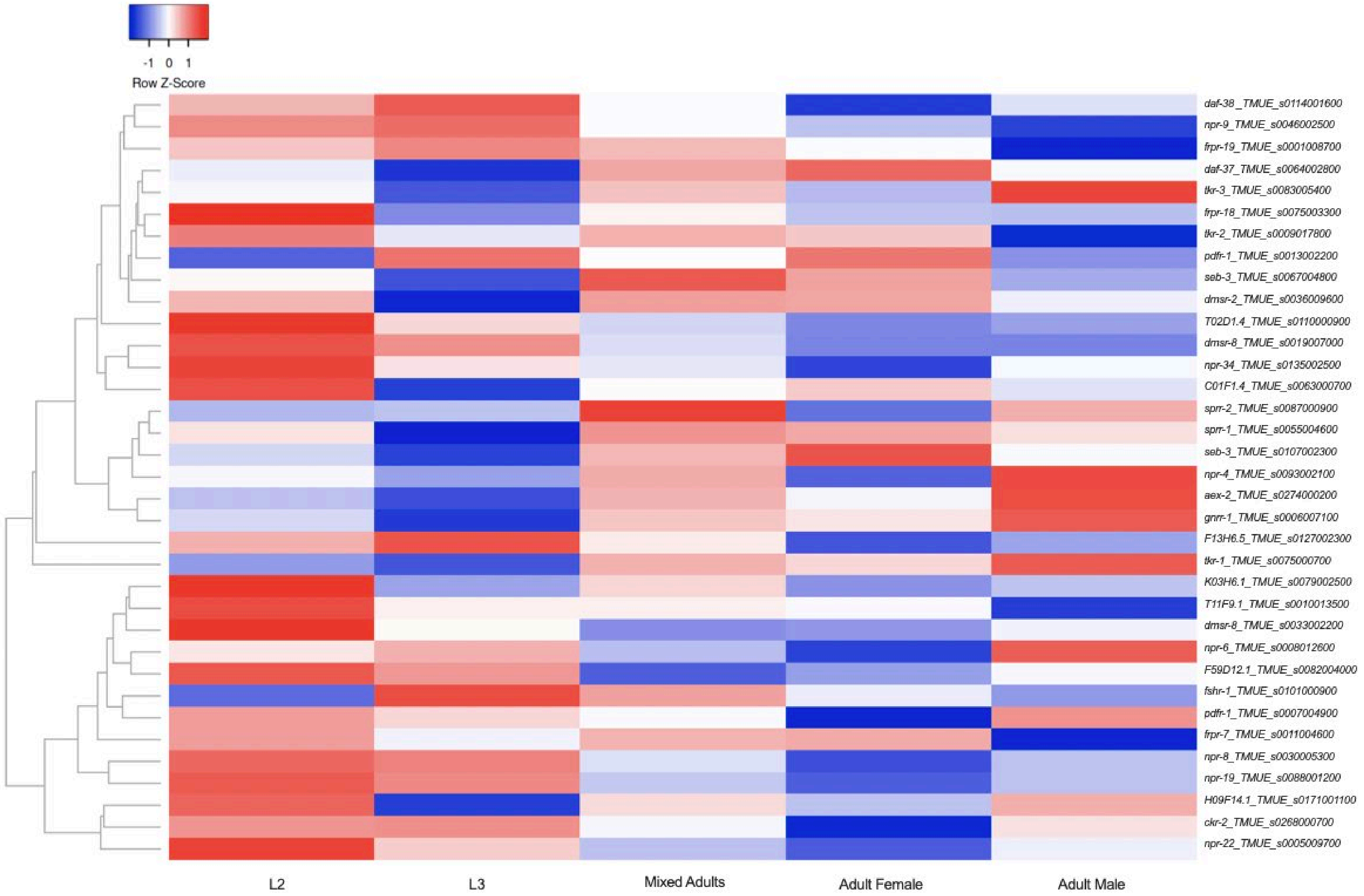

Expression heatmap generated from log2FPKM values of 35 genes in *Trichuris muris* identified from developmentally staged RNA-seq libraries and https://parasite.wormbase.org utilising Heatmapper's Expression program [http://heatmapper.ca/expression, (Babicki et al., 2016)]. Average Clustering Method & Pearson's Distance Measurement Method were used. Life stages are represented in columns (L2, L3, Mixed Adults, Adult Female, Adult Male). Rows indicated individual genes, denoted by the gene ID.

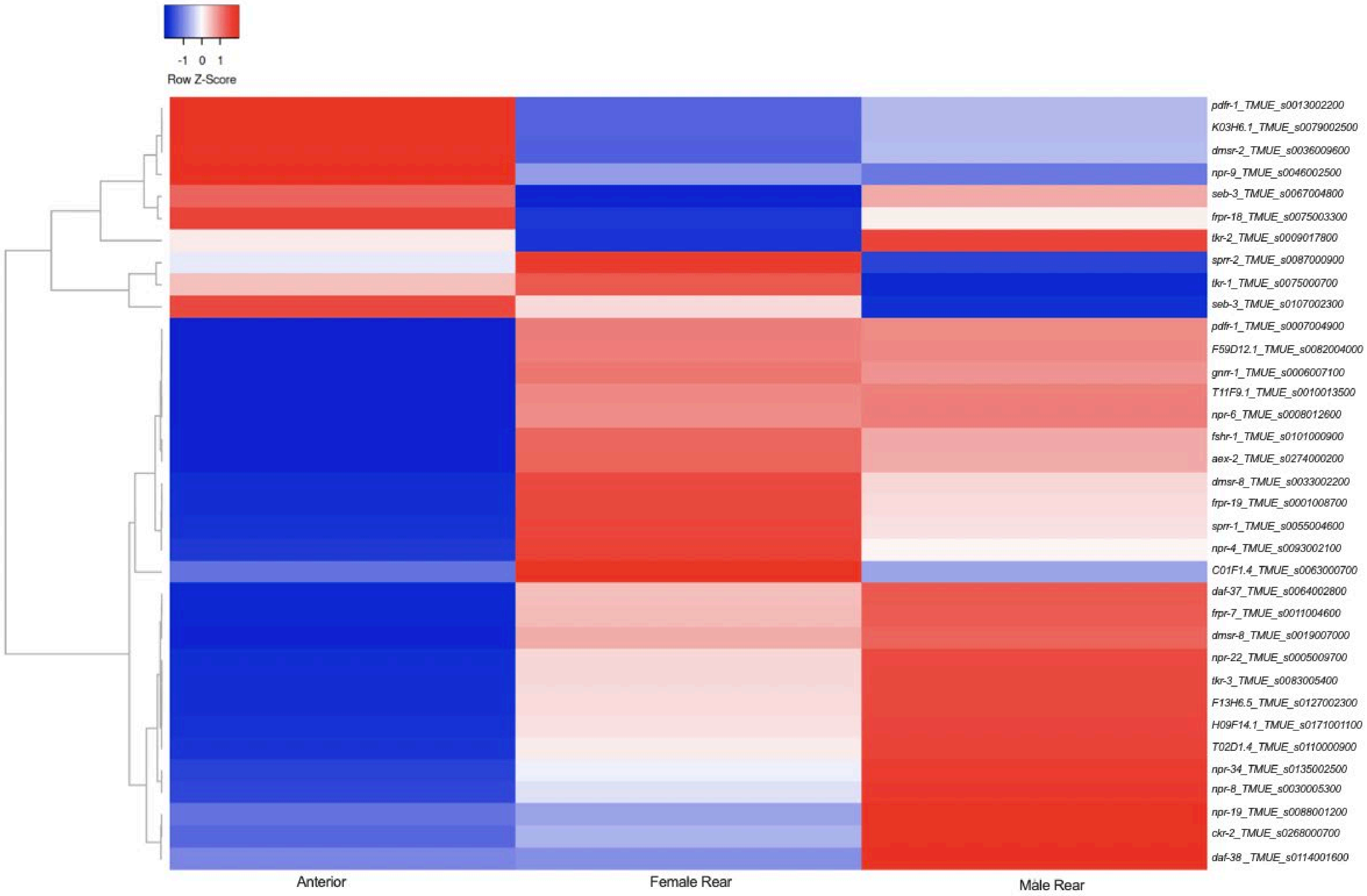

Expression heatmap generated from logFPKM values of 35 genes in *Trichuris muris* identified from developmentally staged RNA-seq libraries and https://parasite.wormbase.org utilising Heatmapper's Expression program [http://heatmapper.ca/expression, (Babicki et al., 2016)]. Average Clustering Method & Pearson's Distance Measurement Method were used. Tissues are represented in columns (Anterior, Female Rear, Male Rear). Rows indicated individual genes, denoted by the gene ID.

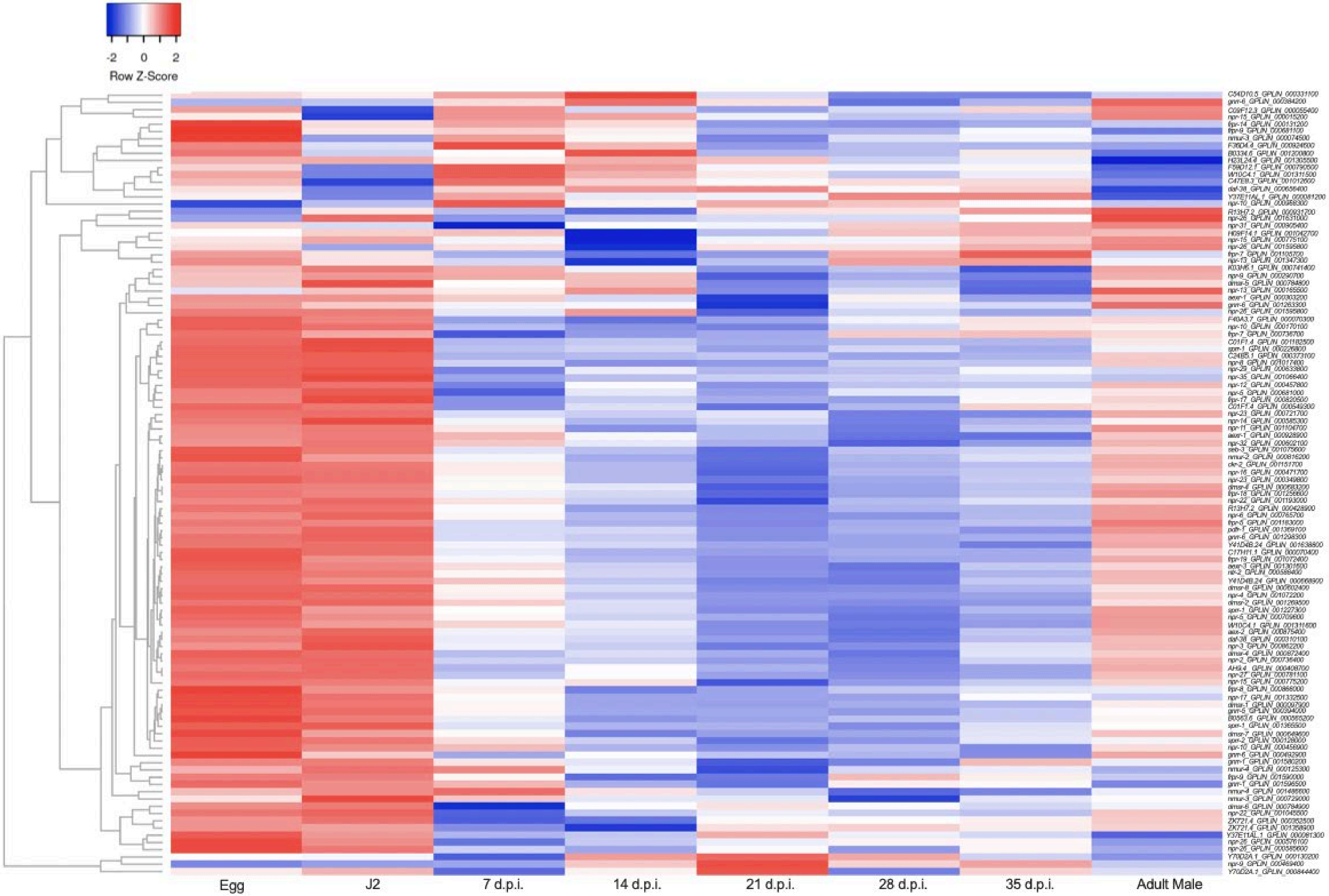

Expression heatmap generated from log2TPM values of 108 genes in *Globodera pallida* identified from developmentally staged RNA-seq libraries and https://parasite.wormbase.org utilising Heatmapper's Expression program [http://heatmapper.ca/expression, (Babicki et al., 2016)]. Average Clustering Method & Pearson's Distance Measurement Method were used. Life stages are represented in columns (Egg, J2, 7 days post infection, 14 days post infection, 21 days post infection, 28 days post infection, 35 days post infection, Adult Male). Rows indicated individual genes, denoted by the gene ID.

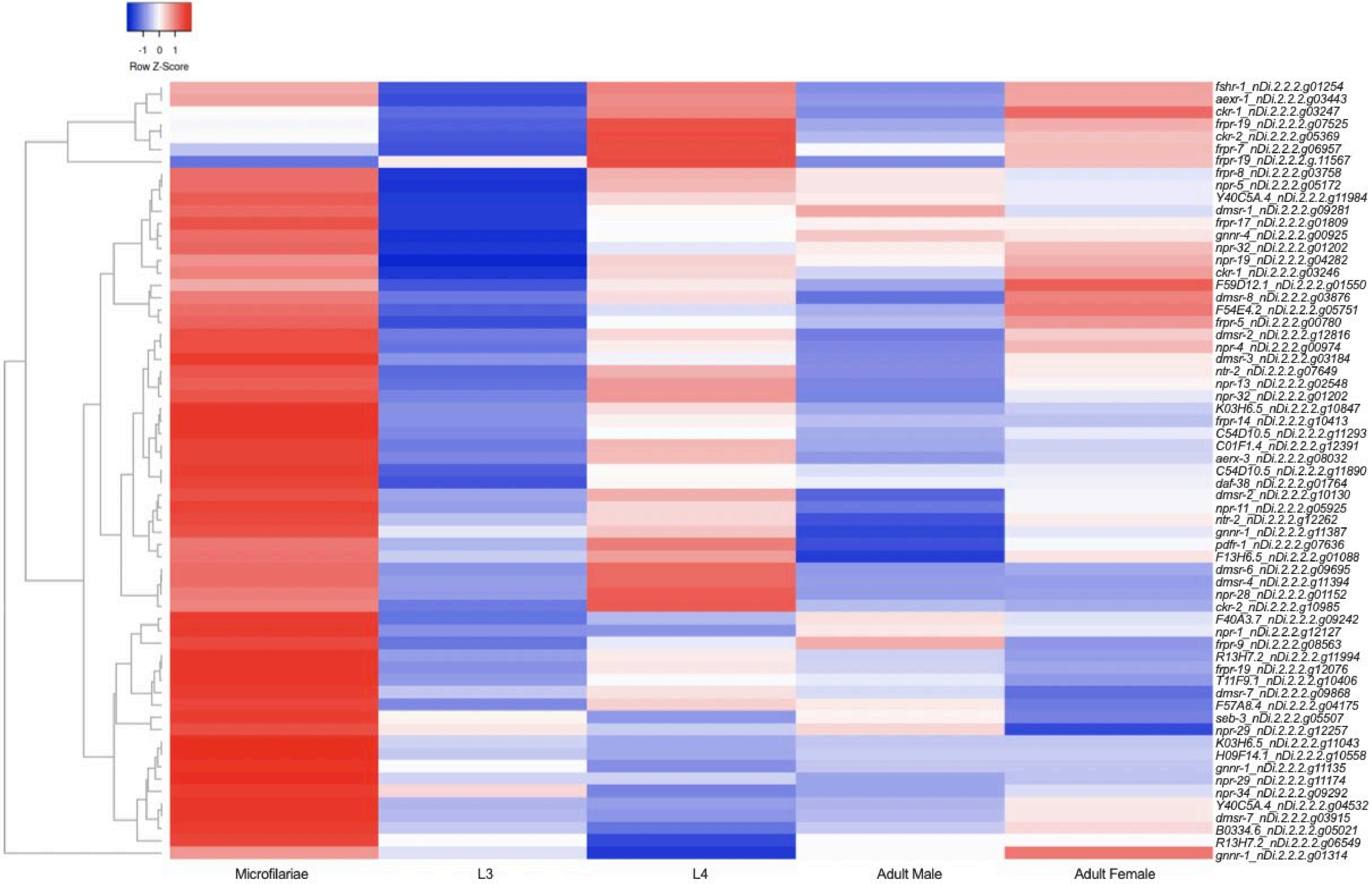

Expression heatmap generated from log2FPKM values of 63 genes in *Dirofilaria immitis* identified from developmentally staged RNA-seq libraries and https://parasite.wormbase.org utilising Heatmapper's Expression program [http://heatmapper.ca/expression, (Babicki et al., 2016)]. Average Clustering Method & Pearson's Distance Measurement Method were used. Life stages are represented in columns (Microfilariae, L3, L4, Adult Male, Adult Female). Rows indicated individual genes, denoted by the gene ID.

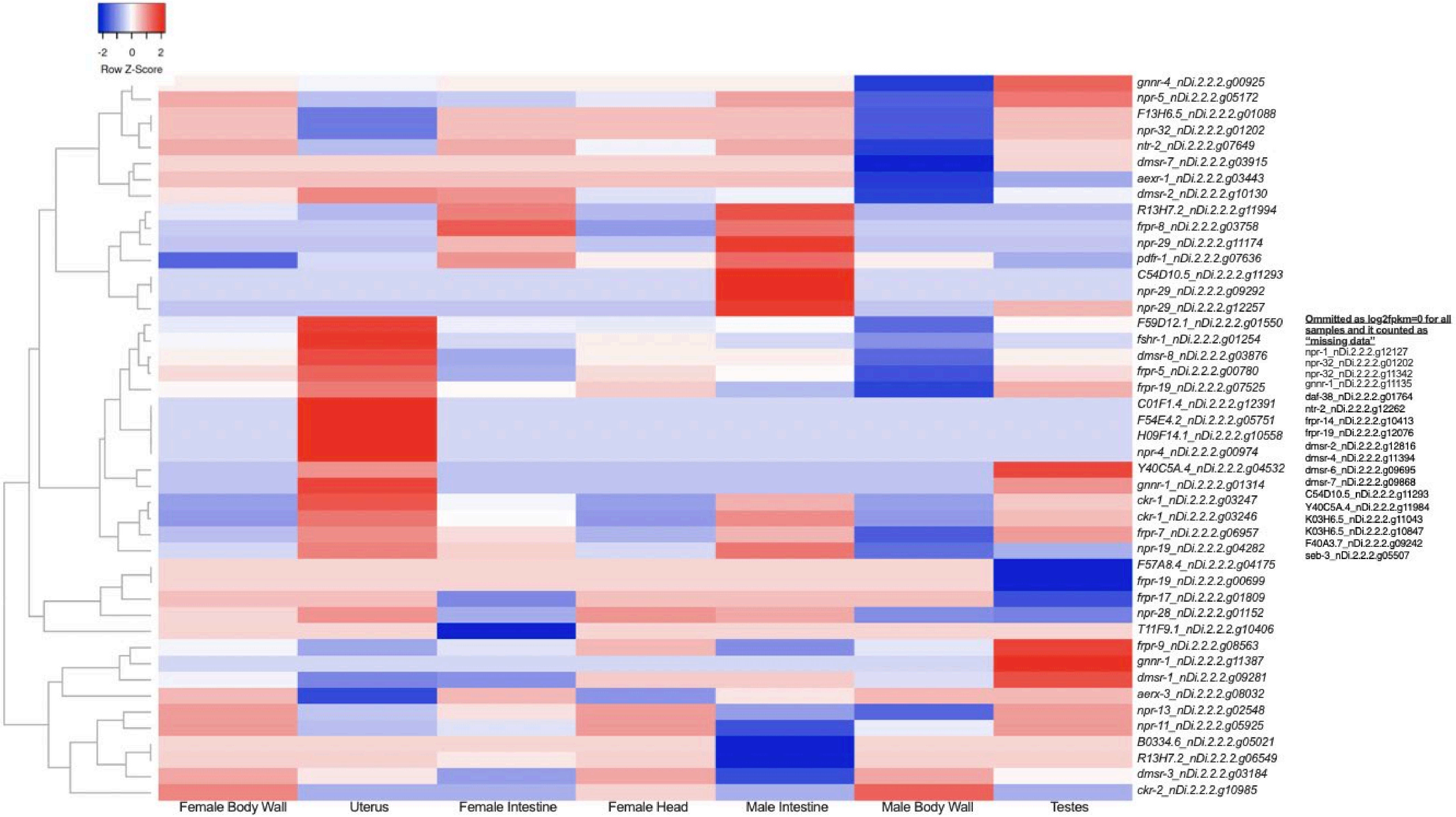

Expression heatmap generated from log2FPKM values of 45 genes in *Dirofilaria immitis* identified from developmentally staged RNA-seq libraries and https://parasite.wormbase.org utilising Heatmapper's Expression program [http://heatmapper.ca/expression, (Babicki et al., 2016)]. Average Clustering Method & Pearson's Distance Measurement Method were used. Tissues are represented in columns (Female Body Wall, Uterus, Female Intestine, Female Head, Male Intestine, Male Body Wall, Testes). Rows indicated individual genes, denoted by the gene ID.

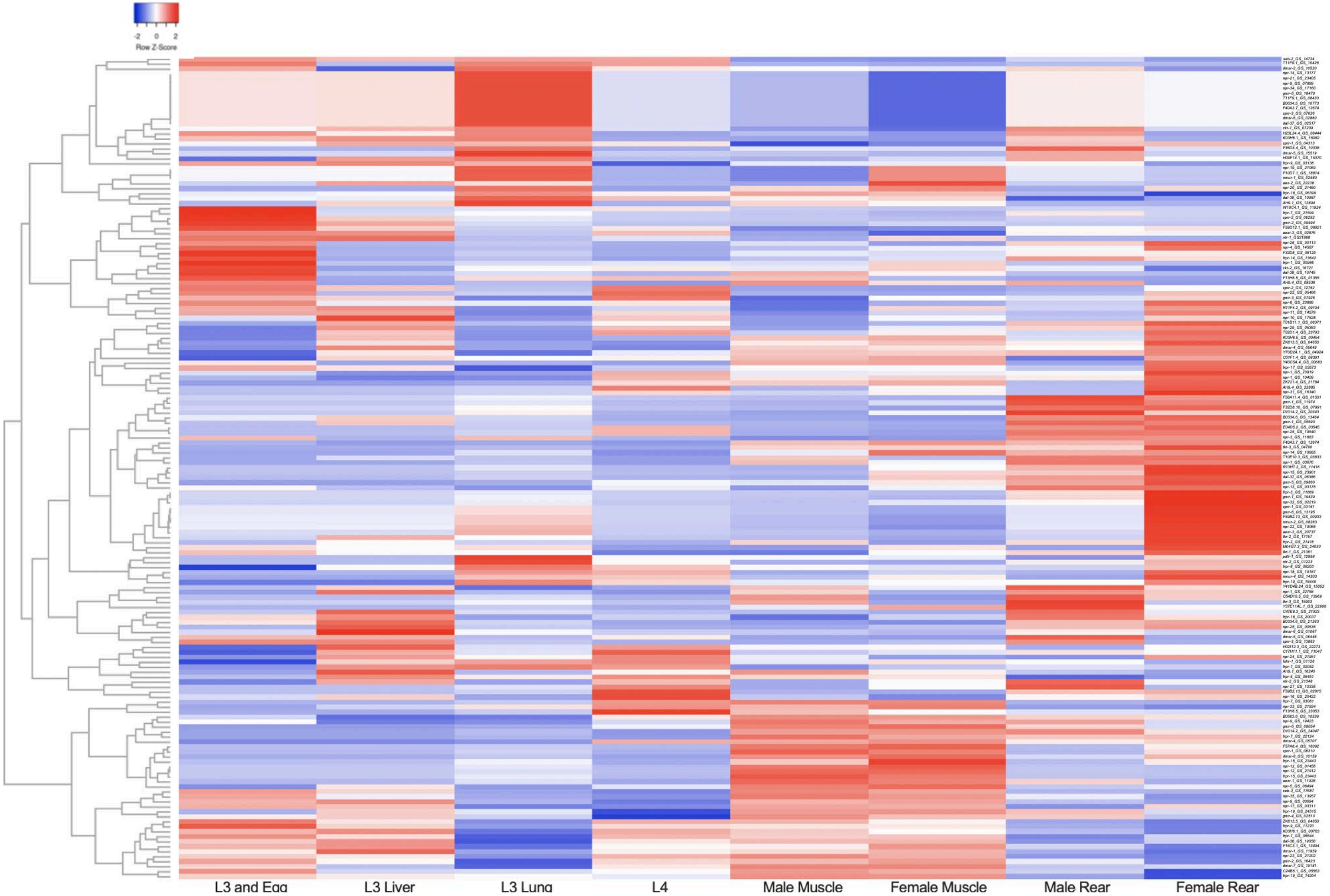

Expression heatmap generated from log2RPKM values of 165 genes in *Ascaris suum* identified from developmentally staged RNA-seq libraries and https://parasite.wormbase.org utilising Heatmapper's Expression program [http://heatmapper.ca/expression, (Babicki et al., 2016)]. Average Clustering Method & Pearson's Distance Measurement Method were used. Samples are represented in columns (L3 and Egg, L3 Liver, L3 Lung, L4, Male Muscle, Female Muscle, Male Rear, Female Rear). Rows indicated individual genes, denoted by the gene ID.

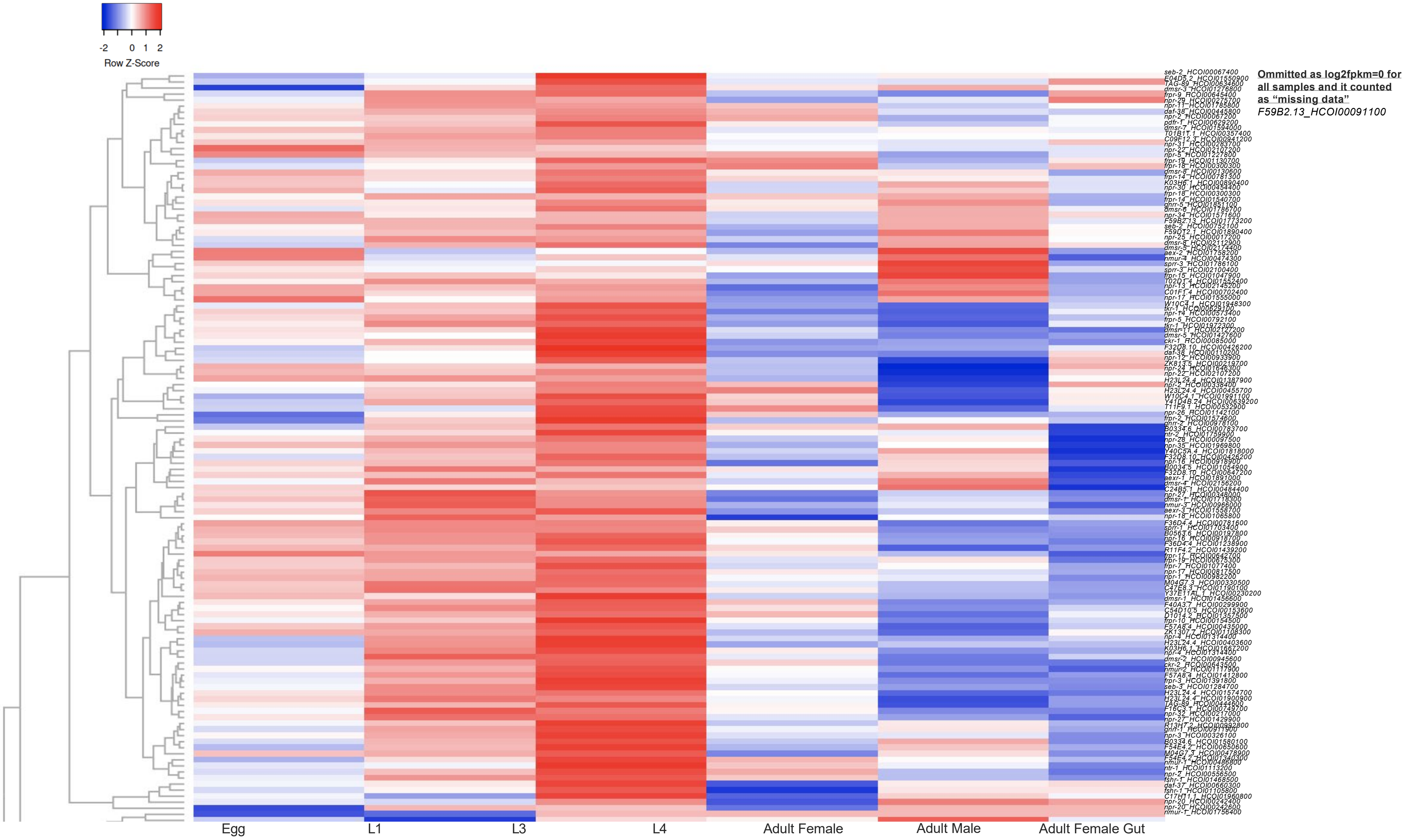

Expression heatmap generated from log2FPKM values of 137 genes in *Haemonchus contortus* identified from developmentally staged RNA-seq libraries and https://parasite.wormbase.org utilising Heatmapper's Expression program [http://heatmapper.ca/expression, (Babicki et al., 2016)]. Average Clustering Method & Pearson's Distance Measurement Method were used. Samples are represented in columns (Egg, L1, L3, L4, Adult Female, Adult Male, Adult Male Gut). Rows indicated individual genes, denoted by the gene ID.
