# Supplementary material for: Phylum-Spanning Neuropeptide GPCR Identification and Prioritization: Shaping Drug Target Discovery Pipelines for Nematode Parasite Control
Source: Front Endocrinol (Lausanne). 2021 Sep 30;12:718363. doi: 10.3389/fendo.2021.718363 (PMC8515059; doi:10.3389/fendo.2021.718363)
Supplement: Supplementary Table 1 — Parasitic nematode species examined in this study. APN, animal parasitic nematode; HPN, human parasitic nematode; PPN, plant parasitic nematode; EPN, entomopathogenic nematode. Genomes and transcriptomes used: Trichuris muris (21), Trichinella spiralis (67), Romanomermis culicivorax (68), Ascaris suum (22, 23), Brugia malayi (24, 25, 69), Dirofilaria immitis (26, 70), Necator americanus (71), Haemonchus contortus (27), Bursaphelenchus xylophilus (72), Globodera pallida (28). [file Table_1.pdf]

Clade	Species	Lifestyle	Genome	Life-stage transcriptome
2	*Trichuris muris*	APN	Foth et al. [21]	Foth et al. [21]
	Trichinella spiralis	HPN	Mitreva et al. [67]	None available/analysed
	Romanomermis culicivorax	EPN	Shiffer et al. [68]	None available/analysed
8	*Ascaris suum*	APN	Jex et al. [22]	Jex et al. [22]; Rosa et al. [23]
	Brugia malayi	HPN	Tracey et al. [69]	Choi et al. [24]; Ballesteros et al. [25]
	Dirofilaria immitis	APN	Godel et al. [70]	Luck et al. [26]
9	*Necator americanus*	HPN	Tang et al. [71]	None available/analysed
	Haemonchus contortus	APN	Laing et al. [27]	Laing et al. [27]
10	*Bursaphelenchus xylophilus*	PPN	Kikuchi et al. [72]	None available/analysed
12	*Globodera pallida*	PPN	Cotton et al. [28]	Cotton et al. [28]
